# Impact of dienogest pretreatment on IVF-ET outcomes in patients with endometriosis: a systematic review and meta-analysis

**DOI:** 10.1186/s13048-023-01245-8

**Published:** 2023-08-16

**Authors:** Wenjing Shao, Yuying Li, Yanli Wang

**Affiliations:** 1 Department of Gynecology, The First Hospital of Jilin University, No.71 Xinmin Avenue, Chaoyang District, Jilin 130021 Changchun, China; 2 Department of Hematology, The First Hospital of Jilin University, No.71 Xinmin Avenue, Chaoyang District, Jilin 130021 Changchun, China

**Keywords:** Dienogest, Endometriosis, IVF-ET, Meta-analysis

## Abstract

**Background:**

To comprehensively evaluate the influence of dienogest (DNG) versus non-DNG pretreatment on in vitro fertilization and embryo transfer (IVF-ET) outcomes for patients with endometriosis.

**Methods:**

PubMed, Embase, Cochrane Library, Web of Science, CNKI, WanFang, and VIP were comprehensively searched for relevant publications until September 14, 2022. Primary outcomes included clinical pregnancy rate and live birth rate. Secondary outcomes included retrieved oocytes, mature oocytes, blastocysts, growing follicles, transferrable embryos, fertilization rate, implantation rate, and miscarriage rate. Subgroup analysis was performed according to different grouping methods and embryo types.

**Results:**

Five studies of 568 females with endometriosis were involved in this systematic review and meta-analysis. DNG treatment exhibited similar effects to non-DNG treatment on either the primary or the secondary outcomes (all *P* > 0.05). The DNG group had a significantly greater clinical pregnancy rate than the non-hormonal treatment group (pooled relative risk [RR]: 2.055, 95% confidence interval [CI]: 1.275, 3.312, *P* = 0.003), and exhibited a significantly lower clinical pregnancy rate than the long gonadotropin-releasing hormone agonist (GnRH-a) group (RR: 0.542, 95%CI: 0.321, 0.916, *P* = 0.022). For patients undergoing fresh embryo transfer, the DNG group displayed a significantly greater clinical pregnancy rate versus the non-DNG group (pooled RR: 1.848, 95%CI: 1.234, 2.767, *P* = 0.003). Patients receiving DNG had a significantly greater live birth rate than those with non-hormonal treatment (pooled RR: 2.136, 95%CI: 1.223, 3.734, *P* = 0.008), while having a significantly lower live birth rate than the long GnRH-a group (RR: 0.441, 95%CI: 0.214, 0.907, *P* = 0.026). While using fresh embryos, patients with DNG treatment had an increased live birth rate, compared with those without DNG treatment (pooled RR: 2.132, 95%CI: 1.090, 4.169, *P* = 0.027).

**Conclusion:**

DNG treatment may have similar effects to non-DNG treatment on IVF-ET outcomes. The clinical pregnancy rate and live birth rate after DNG treatment may be significantly higher than those after non-hormonal treatment. More evidence is warranted to corroborate these findings.

**Supplementary Information:**

The online version contains supplementary material available at 10.1186/s13048-023-01245-8.

## Background

Endometriosis is featured by endometrioid tissue (glands and stroma) being out of the uterus and influences around 15% of women of reproductive age [1]. This disease occurs in up to 50% of infertile women [2], and approximately 30% to 50% of patients with endometriosis suffer from infertility [3], indicating a close relationship between endometriosis and infertility. Infertile females who have endometriosis usually need assisted reproduction technology (ART), including in vitro fertilization and embryo transfer (IVF-ET) [4], to conceive; nevertheless, the IVF-ET success rate among these women is nearly 50% of that among those without endometriosis [5, 6]. Hence, managing endometriosis-related infertility for better ART outcomes is a primary concern of reproductive medicine.

One approach for ART outcome optimization in infertile females who have endometriosis is extended pre-cycle inhibitory hormonotherapy. Down-regulation using a gonadotropin-releasing hormone agonist (GnRH-a) for 3 to 6 months quadrupled the rate of clinical pregnancy among females with endometriosis [7]. GnRH-a administration for 3 months before IVF-ET was shown to improve reproductive outcomes via lessening the deleterious impacts of cytotoxic cytokines and oxidative stress for infertile females who had endometriosis [8]. Dienogest (DNG), a derivative of 19-norsteroids, is greatly selective for progesterone receptor agonists. DNG exerts suppressive effects on endometriotic lesions and cytokines, and was reported to be more cytoreductive than GnRH-a on endometriotic lesions [9, 10]. Additionally, it was demonstrated that clinical pregnancy and live birth rates were greater in women receiving DNG compared with those in women without hormonotherapy [11], while another research showed contrary results that growing follicles, retrieved oocytes, blastocysts, cumulative pregnancy rate, and live birth rate were prominently reduced among women receiving DNG versus GnRH-a [12]. The effect of DNG pretreatment on IVF-ET outcomes for females with endometriosis remains vague.

This study intended to comprehensively assess the influence of DNG treatment versus non-DNG treatment preceding IVF-ET on IVF-ET outcomes among patients with endometriosis via a systematic review and meta-analysis. Subgroup analysis was further conducted based on different grouping methods and embryo types.

## Methods

### Search strategy

PubMed, Embase, Cochrane Library, and Web of Science were comprehensively examined for relevant publications up to September 14, 2022. English search terms were “dienogest” OR “17 alpha-cyanomethyl-17 beta-hydroxy-13 beta-methylgona-4,9-dien-3-one” OR “17 alpha-cyanomethyl-17 beta-hydroxyestra-4,9(10)-diene-3-one” OR “19-norpregna-4,9-diene-21-nitrile, 17-hydroxy-3-oxo-, 17alpha” OR “STS 557” OR “STS-557” OR “Visanne” AND “Fertilization in Vitro” OR “Fertilizations in Vitro” OR “In Vitro Fertilization” OR “In Vitro Fertilizations” OR “Test-Tube Fertilization” OR “Test Tube Fertilization” OR “Test-Tube Fertilizations” OR “Test-Tube Babies” OR “Test Tube Babies” OR “Test-Tube Baby” OR “IVF” OR “Embryo Transfer” OR “Embryo Transfers” OR “Blastocyst Transfer” OR “Tubal Embryo Transfer” OR “Tubal Embryo Stage Transfer” OR “IVF-ET”. This search was conducted by WJ Shao and YL Wang independently, and discussion was needed to resolve disagreements. The current meta-analysis was performed following the Preferred Reporting Items for Systematic Reviews and Meta-Analyses (PRISMA).

### Inclusion and exclusion criteria

Inclusion criteria were as follows: (1) articles on patients with endometriosis; (2) articles with the study group treated with DNG (DNG group), and the control group not treated with DNG (non-DNG group); (3) articles having any of the following outcomes: retrieved oocytes, mature oocytes, blastocysts, growing follicles, transferrable embryos, fertilization rate, implantation rate, clinical pregnancy rate, miscarriage rate, and live birth rate; (4) randomized controlled trials (RCTs) or cohort studies.

Exclusion criteria were as follows: (1) studies involving animal experiments; (2) reviews, meta-analyses, case reports, conference reports, editorial materials, and protocols; (3) non-English studies.

### Outcome measures

Primary outcomes included clinical pregnancy rate and live birth rate. Secondary outcomes included retrieved oocytes, mature oocytes, blastocysts, growing follicles, transferrable embryos, fertilization rate, implantation rate, and miscarriage rate. The clinical pregnancy rate referred to the number of successful clinical pregnancies (gestational sacs and germs observed by B-ultrasonography) divided by the number of transplant cycles. The live birth rate referred to the number of final live births divided by the number of transplant cycles. The miscarriage rate referred to the number of identified intrauterine gestational sacs without fetal poles, or fetal poles without heart pulsations without other viable fetuses divided by the number of transplant cycles.

### Data extraction and quality assessment

The extracted data included first author, year of publication, country, study period, study design, group, interventions, number of included women (N), age (years), body mass index (BMI, kg/m^2^), cyst size (cm), staging, duration of subfertility (years), laterality, surgery, previous treatment, follicle-stimulating hormone (FSH, IU/L), anti-Mullerian hormone (AMH, ng/mL), antral follicle count (AFC), quality assessment, and outcomes.

### Quality and bias assessment

The Cochrane Collaboration’s tool [13] was used to assess the risk of bias in the included RCTs. The domains for assessment included random sequence generation, allocation concealment, blinding of participants and personnel, blinding of outcome assessment, incomplete outcome data, selective reporting, and other bias. The risk of bias was classified into low, unclear or high. The quality of cohort studies was evaluated using the Newcastle–Ottawa Scale (NOS) [14]. The total score of this scale was 9 points (0–3: poor quality, 4–6: fair quality, 7–9: good quality).

### Statistical analysis

All studies were statistically analyzed with Stata 15.1 (Stata Corporation, College Station, TX, USA). Relative risks (RRs) acted as the statistic for enumeration data, weighted mean differences (WMDs) were used as the statistic for measurement data, and effect sizes were illustrated as 95% confidence intervals (CIs). The effect size of each outcome was examined for heterogeneity, and when the heterogeneity statistic I^2^ ≥ 50%, we adopted the random-effects model for analysis; otherwise, we employed the fixed-effects model. Subgroup analysis was further performed according to grouping methods and embryo types. As I^2^ ≥ 50%, meta-regression analysis was conducted to investigate the source of the heterogeneity. Sensitivity analyses were carried out for both the primary and the secondary outcomes. *P* < 0.05 indicated statistical significance.

## Results

### Characteristics of the included studies

**Fig. 1:**
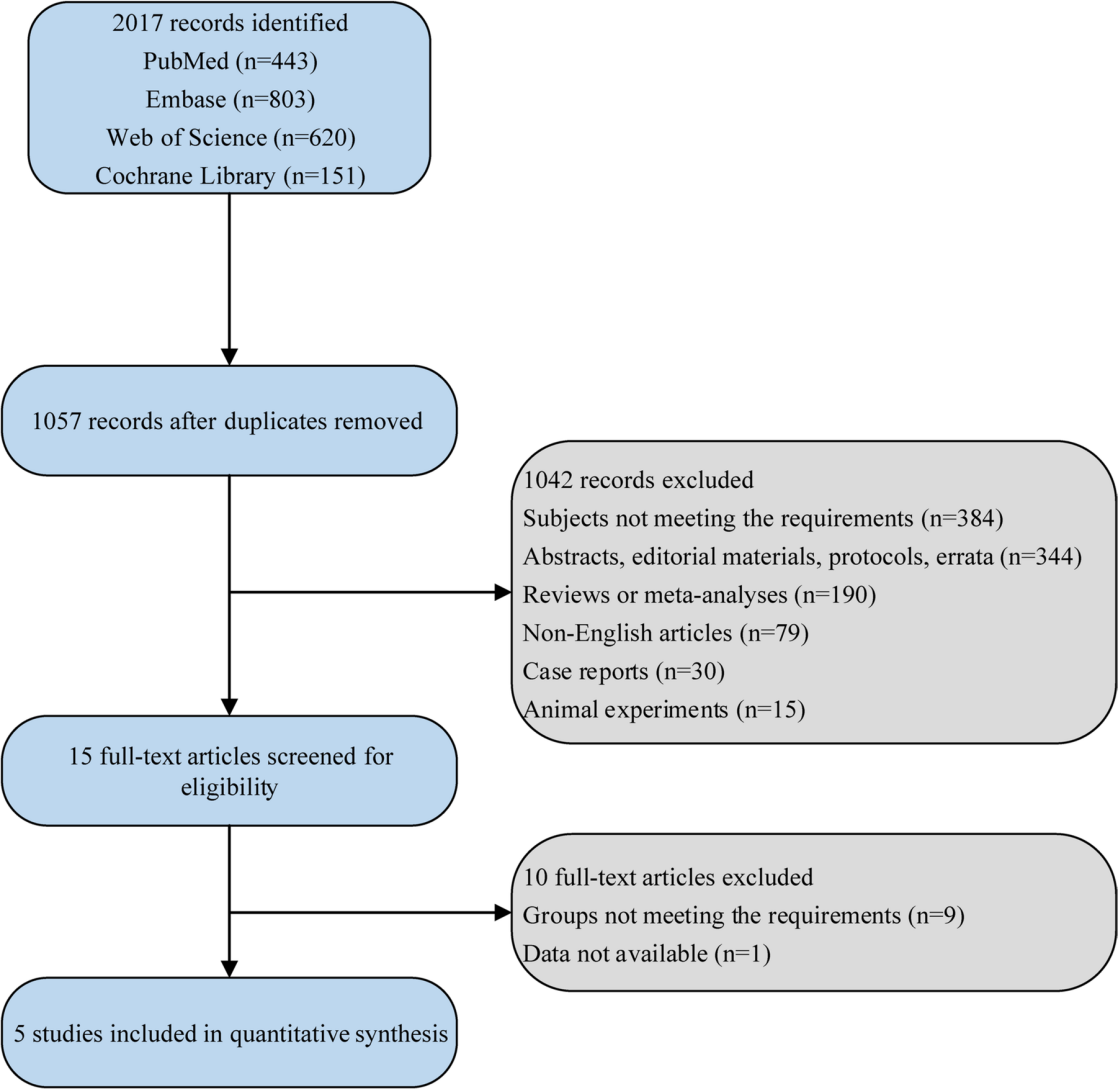
Flow chart for study screening

In total, 2,017 articles were retrieved from the four databases, with 443 from PubMed, 803 from Embase, 620 from Web of Science, and 151 from Cochrane Library. After duplicate removal and according to the eligibility criteria, five studies [11, 12, 15–17] were included for quantitative analysis. The flow chart of study screening is shown in Fig. 1. A total of 568 patients were enrolled, of which 269 were in the DNG group and 299 in the non-DNG group. Table 1 exhibits basic information of the included studies. Three included studies reported the stage of the disease, and two did not provide relevant information on the disease stage. Patients had stage I/II and III/IV endometriosis in the study of Iwami et al. [16]; patients had minimal, mild, moderate, and severe endometriosis in the study of Khalifa et al. [17]; patients had III and IV endometriosis in the study of Tamura et al. [12]. Regarding surgery, three studies had relevant information: patients in the study of Iwami et al. [16] underwent laparoscopic surgery or laparotomy for ovarian endometrial cysts, patients in the study of Barra et al. [15] did not had operations, and patients in the study of Muller et al. [11] underwent laparoscopic surgery of ovarian endometriomas before IVF. The other two studies did not report information on surgery.

**Table 1:** The basic characteristics of the included studies

Author	Year	Country	Period	Study design	Group	Interventions	N	Age (years)	BMI (kg/m^2^)	Cyst size (cm)	Staging	Duration of subfertility (years)	Laterality	Surgery	Previous treatment	FSH (IU/L)	AMH (ng/mL)	AFC	Outcomes
Iwami	2021	Japan	2018.02–2020.05	Prospective cohort	DNG	Severe dysmenorrhea or a high risk of endometriosis recurrence following previous surgery take 2 mg of DNG daily prior to ART, continue DNG and start the COH procedure at individual discretion	71	35.0 ± 4.31	21.9 ± 3.6	-	I/II 46, III/IV 25	3.9 ± 2.5	-	58	-	7.82 ± 2.53	2.67 ± 1.92	7.21 ± 3.71	Mature oocytes, growing follicles, fertilization rate, implantation rate, clinical pregnancy rate, miscarriage rate, live birth rate
					Dydrogesterone	Dydrogesterone 20 mg/day taken internally, start simultaneously with ovarian stimulation on the second or third days of menstrual cycles	74	34.9 ± 3.71	21.7 ± 2.5	-	I/II 59, III/IV 15	2.6 ± 2.6	-	53	-	7.63 ± 3.05	3.48 ± 1.99	10.70 ± 6.24	
Khalifa	2021	Egypt	2018.08–2019.10	Parallel-group open-label RCT	DNG + short-acting GnRH-a	DNG 2 mg tablet orally daily for 3 months, in the last 3 weeks of the pretreatment period, short-acting GnRH-a in a dose of 0.5 mg SC daily followed by ovarian stimulation when DNG was stopped	67	36.1 ± 2.7	22.3 ± 1.9	-	Minimal 8, mild 12, moderate 22, severe 25	7.2 ± 1.7	-	-	ART 17	4.3 ± 1.8	2.8 ± 1.8	11.8 ± 2.3	Retrieved oocytes, mature oocytes, transferrable embryos, fertilization rate, clinical pregnancy rate, miscarriage rate
					GnRH-a ultra-long protocol	GnRH-a in the form of depot leuprorelin acetate 3.75 mg SC monthly injections for 3 months before starting ovarian stimulation after the third dose of depot GnRH-a	67	35.6 ± 3.5	22.5 ± 1.6	-	Minimal 5, mild 13, moderate 27, severe 22	6.6 ± 1.5	-	-	ART 20	5.4 ± 1.7	3.5 ± 1.3	12 ± 3.2	
Barra	2020	Italy	2015.06–2019.09	Retrospective cohort	DNG	A 3-month treatment with DNG (2 mg daily) before IVF	63	36.4 ± 4.0	20.7 ± 2.8	2.7 ± 2.0	-	-	Left 22, right 16	0	Hormonal therapy for endometriosis 37, failed embryo Transfers 1.4 ± 0.5	7.8 ± 2.2	2.6 ± 0.8	6.6 ± 3.2	Mature oocytes, growing follicles, blastocysts, transferrable embryos, implantation rate, clinical pregnancy rate, live birth rate
					Without hormonal treatment	Direct IVF	88	36.2 ± 4.1	21.0 ± 3.0	3.6 ± 1.8	-	-	Left 25, right 24	0	Hormonal therapy for endometriosis 55, failed embryo transfers 1.6 ± 0.4	8.2 ± 2.2	2.6 ± 0.9	6.5 ± 2.8	
Tamura	2019	Japan	2011.02–2015.11	Prospective RCT	DNG	DNG 2 mg orally daily for 3 months before IVF	30	34.2 ± 3.4	-	-	III 10, IV 6	-	-	-	-	-	-	-	Retrieved oocytes, mature oocytes, growing follicles, blastocysts, fertilization rate, implantation rate, clinical pregnancy rate, miscarriage rate, live birth rate
					GnRH-a long protocol	Nasal spray GnRH-a (900 μg/day buserelin acetate) from the mid-luteal phase in the previous cycle to the time of HCG injection for the ovulation induction of the IVF-ET cycle	34	33.6 ± 3.6	-	-	III 9, IV 5	-	-	-	-	-	-	-	
Muller	2017	Russia	2012.01–2015.12	Prospective cohort	DNG	DNG 2 mg daily for 6 months prior to IVF protocol	38	23–42	< 30	Diameter ≤ 3 13, diameter > 3 25	-	-	Unilateral 23, Bilateral 15	38	-	8.89 ± 4.89	1.12 ± 0.41	-	Clinical pregnancy rate, live birth rate
					Without hormonal treatment	Direct IVF	36			Diameter ≤ 3 13, diameter > 3 23	-	-	Unilateral 20, Bilateral 16	36	-	8.71 ± 2.96	1.07 ± 0.59	-	

*AFC* Antral follicle count, *AMH* Anti-Mullerian hormone, *ART* Assisted reproductive technology, *IVF* In vitro fertilization, *BMI* Body mass index, *COH* Controlled ovarian hyperstimulation, *DNG* Dienogest, *FSH* Follicle-stimulating hormone, *GnRH-a* Gonadotropin-releasing hormone agonist, *HCG* Human chorionic gonadotropin, *MRI* Magnetic resonance imaging, *RCT* Randomized controlled trial

### Quality and bias of the included studies

**Fig. 2:**
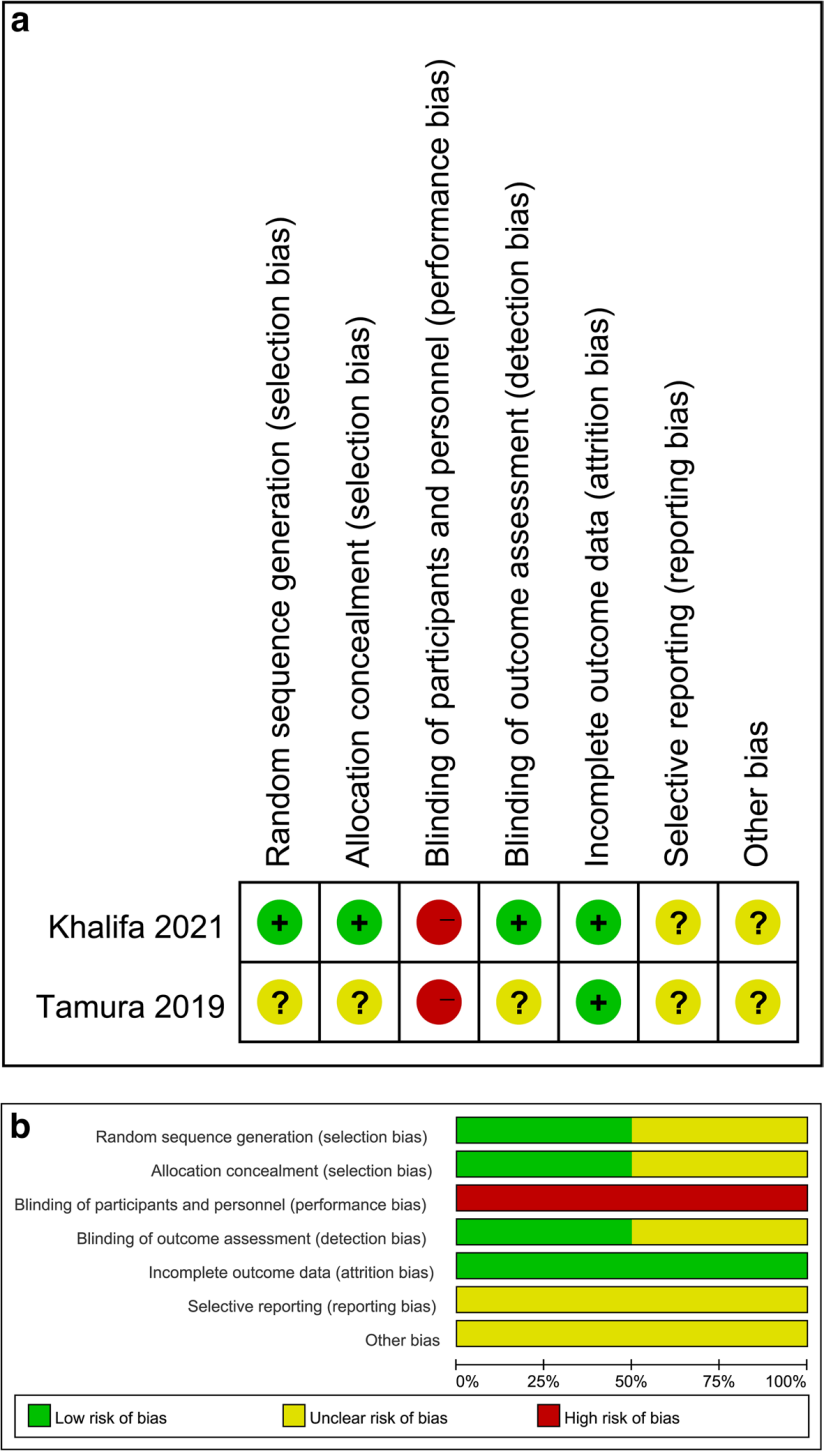
Risk of bias summary and graph: judgements about each risk-of-bias item in the included studies 2a, Risk of bias summary; 2b, Risk of bias graph.

Two included studies were RCTs, and three studies were cohort studies. Regarding the two RCTs, for random sequence generation, allocation concealment, and blinding of outcome assessment, one study has a low risk of bias, and the other study has an unclear risk of bias. Both RCTs had a low risk of bias in incomplete outcome data, an unclear risk of bias in selective reporting and other bias, and a high risk of bias in blinding of participants and personnel. The overall assessment exhibited a moderate level of bias (Fig. 2). For the three cohort studies, two had fair quality, and one had good quality (Table 2).

**Table 2:** Quality assessment of cohort studies using the NOS

Study	Selection				Comparability	Outcome		
	Representativeness of exposed cohort	Selection of non-exposed cohort	Ascertainment of exposure	Demonstration that outcome of interest was not present at start of study	Comparability of cohorts on the basis of the design or analysis	Assessment of outcome	Follow-up long enough	Adequacy of follow up of cohorts
Iwami 2021	*	*	-	*	*	-	*	*
Barra 2020	*	*	-	*	*	-	*	*
Muller 2017	*	*	*	*	*	*	*	-

NOS, Newcastle–Ottawa Scale

### Primary outcomes

#### Clinical pregnancy rate

**Fig. 3:**
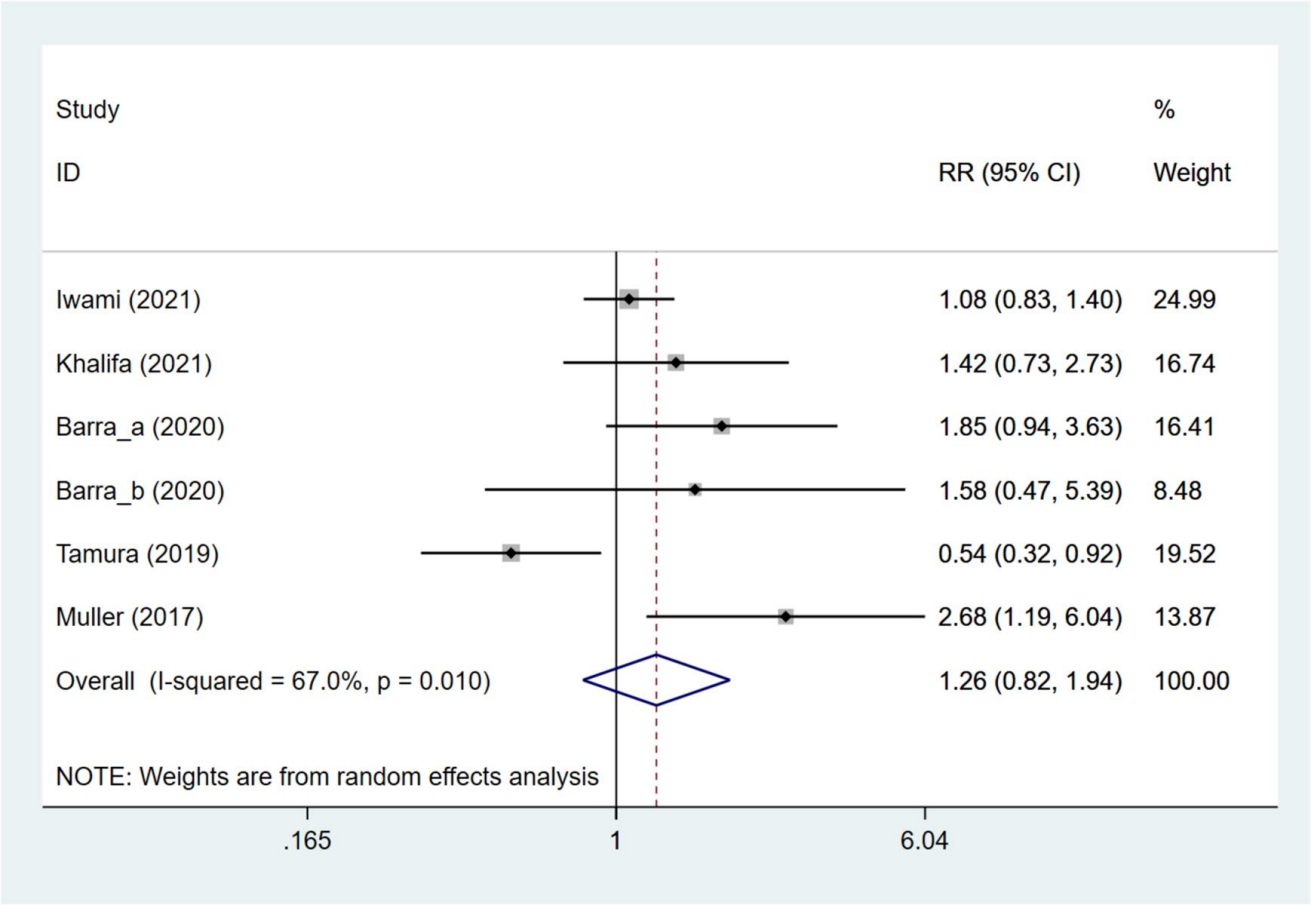
Forest plot for clinical pregnancy rate in the DNG group versus non-DNG group DNG, dienogest; RR, relative risk; CI, confidence interval.

Five studies [11, 12, 15–17] provided data on the clinical pregnancy rate. Combined analysis demonstrated that the DNG group had a comparable clinical pregnancy rate to the non-DNG group (pooled RR: 1.264, 95%CI: 0.824, 1.939, *P* = 0.284) (Fig. 3). Further, the DNG group vs. non-DNG group was divided into DNG group vs. non-hormonal treatment group, DNG + short-acting GnRH-a group vs. ultra-long GnRH-a group, DNG group vs. long GnRH-a group, and DNG group vs. dydrogesterone group, according to different grouping methods. The DNG group was illustrated to have a significantly higher clinical pregnancy rate than the non-hormonal treatment group (pooled RR: 2.055, 95%CI: 1.275, 3.312, *P* = 0.003), and have a significantly lower clinical pregnancy rate versus the long GnRH-a group (RR: 0.542, 95%CI: 0.321, 0.916, *P* = 0.022). No significant difference was identified between the DNG + short-acting GnRH-a group and the ultra-long GnRH-a group (RR: 1.417, 95%CI: 0.735, 2.732, *P* = 0.299), and between the DNG group and the dydrogesterone group (RR: 1.078, 95%CI: 0.827, 1.404, *P* = 0.579). Besides, embryos were classified into frozen and fresh embryos. For patients using frozen embryos, DNG treatment exhibited a similar effect on the clinical pregnancy rate to non-DNG treatment (pooled RR: 1.109, 95%CI: 0.854, 1.439, *P* = 0.438). As for patients undergoing fresh embryo transfer, the DNG group displayed a significantly increased clinical pregnancy rate in contrast to the non-DNG group (pooled RR: 1.848, 95%CI: 1.234, 2.767, *P* = 0.003) (Table 3). Meta-regression analysis showed that heterogeneity in the clinical pregnancy rate did not come from grouping methods and embryo types (all *P* > 0.05) (Table S1).

**Table 3:** Overall and subgroup analyses for the impact of dienogest pretreatment on IVF-ET outcomes in patients with endometriosis

Outcome	Overall/subgroup analysis	Number of studies	RR/WMD (95%CI)	*P*	I^2^
Clinical pregnancy rate	Overall	5	1.264 (0.824, 1.939)	0.284	67.0
	Group				
	DNG vs. no treatment	2	2.055 (1.275, 3.312)	0.003	0.0
	DNG + short-acting GnRH-a vs. ultra-long GnRH-a	1	1.417 (0.735, 2.732)	0.299	NA
	DNG vs. long GnRH-a	1	0.542 (0.321, 0.916)	0.022	NA
	DNG vs. dydrogesterone	1	1.078 (0.827, 1.404)	0.579	NA
	Embryo				
	Frozen	2	1.109 (0.854, 1.439)	0.438	0.0
	Fresh	3	1.848 (1.234, 2.767)	0.003	0.0
Live birth rate	Overall	4	1.234 (0.659, 2.309)	0.511	70.4
	Group				
	DNG vs. no treatment	2	2.136 (1.223, 3.734)	0.008	0.0
	DNG vs. long GnRH-a	1	0.441 (0.214, 0.907)	0.026	NA
	DNG vs. dydrogesterone	1	0.942 (0.661, 1.343)	0.741	NA
	Embryo				
	Frozen	2	1.112 (0.537, 2.305)	0.775	27.0
	Fresh	2	2.132 (1.090, 4.169)	0.027	18.6
Retrieved oocytes	Overall	2	-1.195 (-3.314, 0.923)	0.269	79.3
	Group				
	DNG + short-acting GnRH-a vs. ultra-long GnRH-a	1	-0.300 (-0.742, 0.142)	0.183	NA
	DNG vs. long GnRH-a	1	-2.500 (-4.411, -0.589)	0.01	NA
Mature oocytes	Overall	4	-0.990 (-2.264, 0.285)	0.128	87.3
	Group				
	DNG vs. no treatment	1	0.700 (0.015, 1.385)	0.045	NA
	DNG + short-acting GnRH-a vs. ultra-long GnRH-a	1	-0.600 (-1.132, -0.068)	0.027	NA
	DNG vs. long GnRH-a	1	-2.500 (-4.217, -0.783)	0.004	NA
	DNG vs. dydrogesterone	1	-2.270 (-3.620, -0.920)	0.001	NA
Blastocysts	Overall	2	-1.329 (-3.284, 0.626)	0.183	89.9
	Group				
	DNG vs. no treatment	1	-0.400 (-0.881, 0.081)	0.103	NA
	DNG vs. long GnRH -a	1	-2.400 (-3.549, -1.251)	< 0.001	NA
Transferrable embryos	Overall	2	0.187 (-0.487, 0.862)	0.586	71.3
	Group				
	DNG vs. no treatment	1	-0.100 (-0.420, 0.220)	0.076	NA
	DNG + short-acting GnRH-a vs. ultra-long GnRH-a	1	0.600 (-0.062, 1.262)	0.540	NA
Fertilization rate	Overall	3	1.029 (0.958, 1.105)	0.429	0.0
	Group				
	DNG + short-acting GnRH-a vs. ultra-long GnRH-a	1	1.185 (0.807, 1.740)	0.386	NA
	DNG vs. long GnRH-a	1	0.788 (0.525, 1.185)	0.253	NA
	DNG vs. dydrogesterone	1	1.032 (0.961, 1.108)	0.386	0.0
	Embryo				
	Frozen	1	1.032 (0.961, 1.108)	0.386	0.0
	Fresh	1	1.185 (0.807, 1.740)	0.386	NA
Implantation rate	Overall	3	1.111 (0.888, 1.391)	0.356	42.6
	Group				
	DNG vs. no treatment	1	1.657 (1.012, 2.715)	0.045	0.0
	DNG vs. long GnRH-a	1	0.708 (0.387, 1.297)	0.264	NA
	DNG vs. dydrogesterone	1	1.064 (0.807, 1.403)	0.662	NA
	Embryo				
	Frozen	2	1.084 (0.830, 1.415)	0.553	0.0
	Fresh	1	1.849 (1.031, 3.318)	0.039	NA
Miscarriage rate	Overall	3	1.384 (0.824, 2.325)	0.220	6.1
	Group				
	DNG + short-acting GnRH-a vs. ultra-long GnRH-a	1	0.178 (0.009, 3.433)	0.253	NA
	DNG vs. long GnRH-a	1	1.455 (0.512, 4.134)	0.482	NA
	DNG vs. dydrogesterone	1	1.639 (0.873, 3.078)	0.124	NA
	Embryo				
	Frozen	1	1.639 (0.873, 3.078)	0.124	NA
	Fresh	1	0.178 (0.009, 3.433)	0.253	NA

*DNG* Dienogest, *GnRH-a* Gonadotropin-releasing hormone agonist, *RR* Relative risk, *WMD* Weighted mean difference, *CI* Confidence interval, *NA* Not available

### Live birth rate

**Fig. 4:**
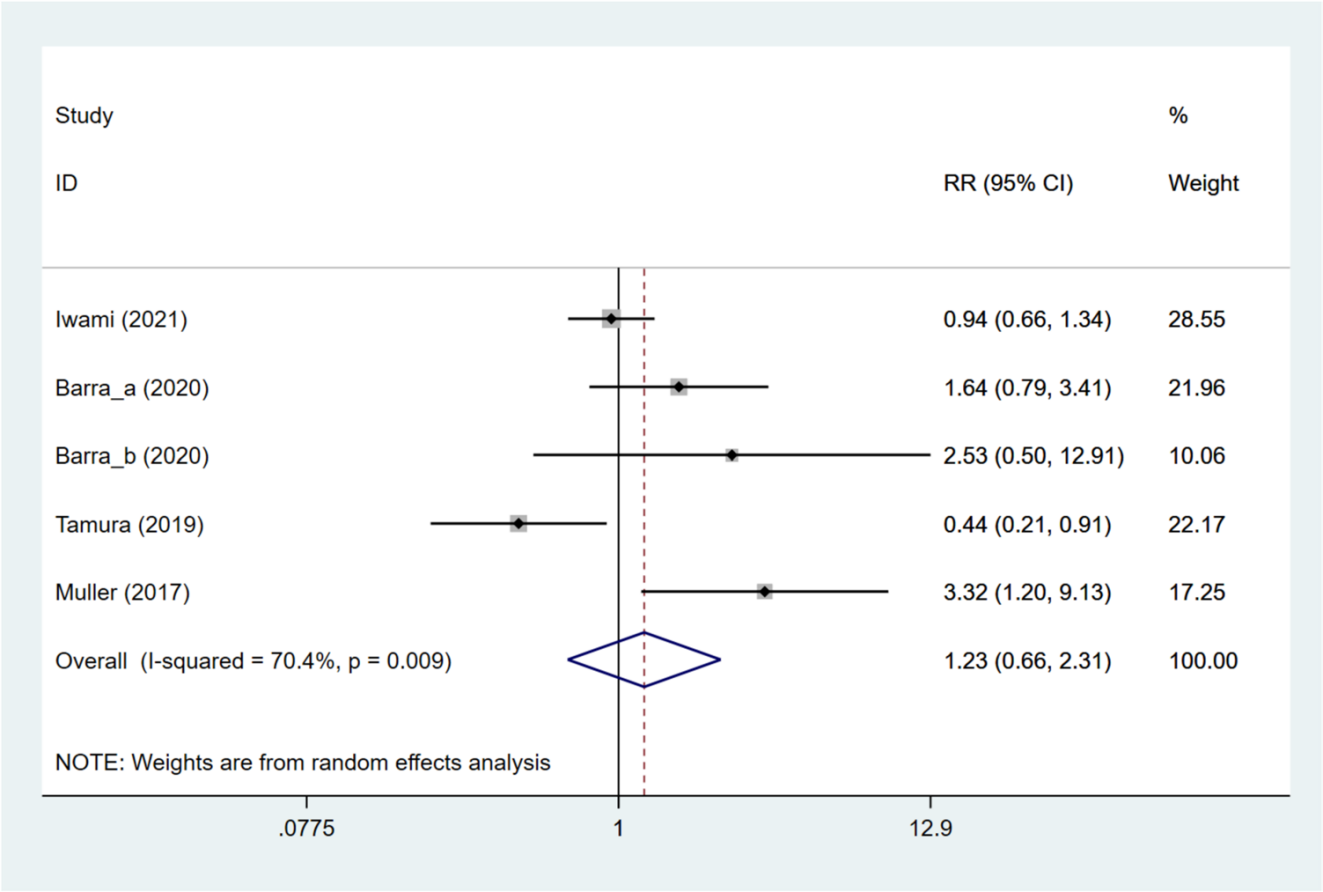
Forest plot for live birth rate in the DNG group versus non-DNG group DNG, dienogest; RR, relative risk; CI, confidence interval.

The live birth rate was studied in four articles [11, 12, 15, 16]. Patients treated with DNG were found to have a comparable live birth rate to those not treated with DNG (pooled RR: 1.234, 95%CI: 0.659, 2.309, *P* = 0.511) based on overall analysis (Fig. 4). Subgroup analysis based on different grouping methods (DNG group vs. non-hormonal treatment group, DNG group vs. long GnRH-a group, and DNG group vs. dydrogesterone group) suggested that the DNG group had a significantly greater live birth rate than the non-hormonal treatment group (pooled RR: 2.136, 95%CI: 1.223, 3.734, *P* = 0.008), and the DNG group had a significantly reduced live birth rate compared with the long GnRH-a group (RR: 0.441, 95%CI: 0.214, 0.907, *P* = 0.026). Comparable live birth rates were observed in the DNG and dydrogesterone groups (RR: 0.942, 95%CI: 0.661, 1.343, *P* = 0.741). While using frozen embryos, no significant difference existed between patients receiving and not receiving DNG treatment (pooled RR: 1.112, 95%CI: 0.537, 2.305, *P* = 0.775). While using fresh embryos, patients with DNG treatment had an increased live birth rate, compared with those without DNG treatment (pooled RR: 2.132, 95%CI: 1.090, 4.169, *P* = 0.027) (Table 3). As meta-regression analysis illustrated, grouping methods and embryo types were not the sources of heterogeneity in the live birth rate (all *P* > 0.05) (Table S1).

### Secondary outcomes

#### Retrieved oocytes

**Fig. 5:**
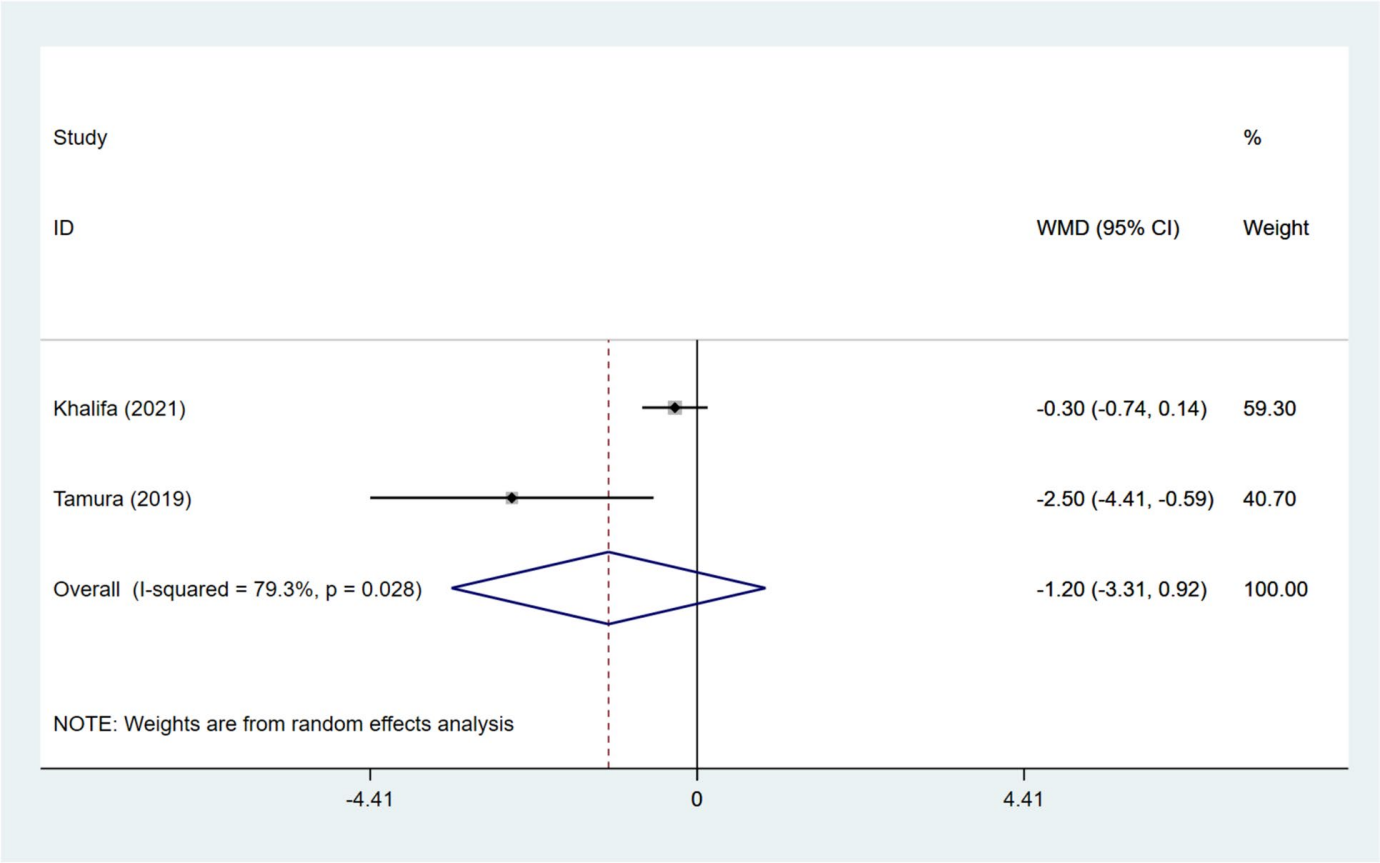
Forest plot for retrieved oocytes in the DNG group versus non-DNG group DNG, dienogest; WMD, weighted mean difference; CI, confidence interval.

Two studies [12, 17] assessed the influence of DNG pretreatment on the number of retrieved oocytes. Overall analysis showed that the DNG and non-DNG groups had the comparable number of retrieved oocytes (pooled WMD: -1.195, 95%CI: -3.314, 0.923, *P* = 0.269) (Fig. 5). Subgroup analysis based on grouping approaches exhibited that the number of retrieved oocytes was similar in the DNG + short-acting GnRH-a group and the ultra-long GnRH-a group (WMD: -0.300, 95%CI: -0.742, 0.142, *P* = 0.183), and the DNG group had fewer retrieved oocytes than the long GnRH-a group (WMD: -2.500, 95%CI: -4.411, -0.589, *P* = 0.01) (Table 3).

### Mature oocytes

**Fig. 6:**
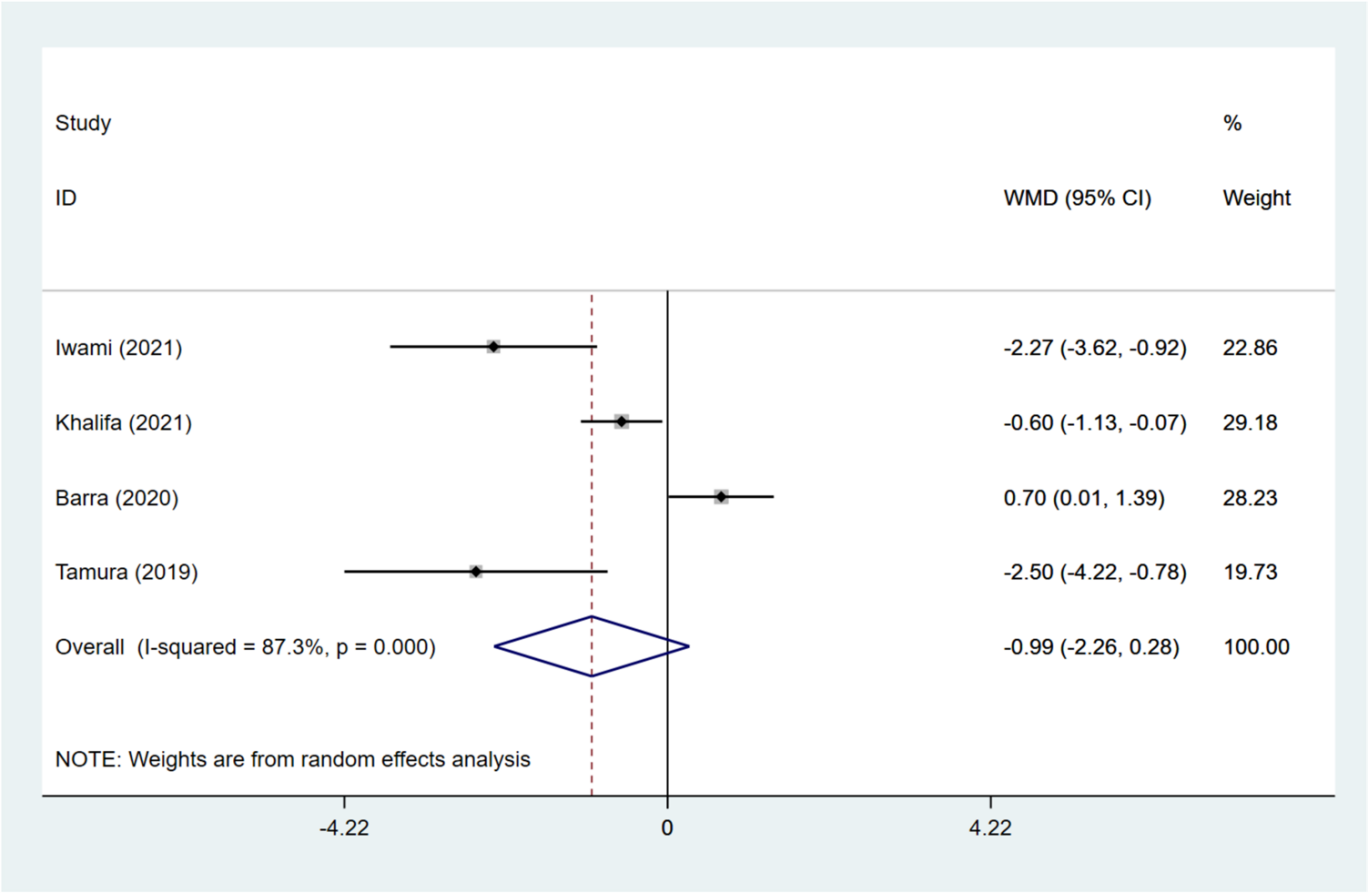
Forest plot for mature oocytes in the DNG group versus non-DNG group DNG, dienogest; WMD, weighted mean difference; CI, confidence interval.

The number of mature oocytes was reported in four studies [12, 15–17]. The number of mature oocytes in the DNG group was equivalent to that in the non-DNG group (pooled WMD: -0.990, 95%CI: -2.264, 0.285, *P* = 0.128) (Fig. 6). In accordance with different grouping methods, the DNG group was demonstrated to have more mature oocytes than the non-hormonal treatment group (WMD: 0.700, 95%CI: 0.015, 1.385, *P* = 0.045). Patients with DNG + short-acting GnRH-a treatment had a smaller number of mature oocytes than those receiving ultra-long GnRH-a (WMD: -0.600, 95%CI: -1.132, -0.068, *P* = 0.027). In contrast to the long GnRH-a group, the DNG group showed reduced mature oocytes (WMD: -2.500, 95%CI: -4.217, -0.783, *P* = 0.004). Patients treated with DNG were found to have fewer mature oocytes than those treated with dydrogesterone (WMD: -2.270, 95%CI: -3.620, -0.920, *P* = 0.001) (Table 3).

### Growing follicles

Three studies [12, 15, 16] provided relevant information on growing follicles. Tamura et al. [12] defined growing follicles as follicles with a diameter ≥ 15 mm, and found that growing follicles were significantly fewer in the DNG group than those in the long GnRH-a group. Iwami et al. [16] defined growing follicles as follicles with a diameter > 10 mm, and demonstrated that the mean number of growing follicles on the trigger day was significantly smaller in the DNG group than that in the dydrogesterone group. Barra et al. [15] reported that DNG pretreatment significantly increased co-dominant follicles (average diameter > 15 mm) than non-hormonal treatment in patients with endometriomas of a diameter ≥ 4 cm (7.3 ± 1.9 vs 5.7 ± 2.3, *P* = 0.036).

### Blastocysts

**Fig. 7:**
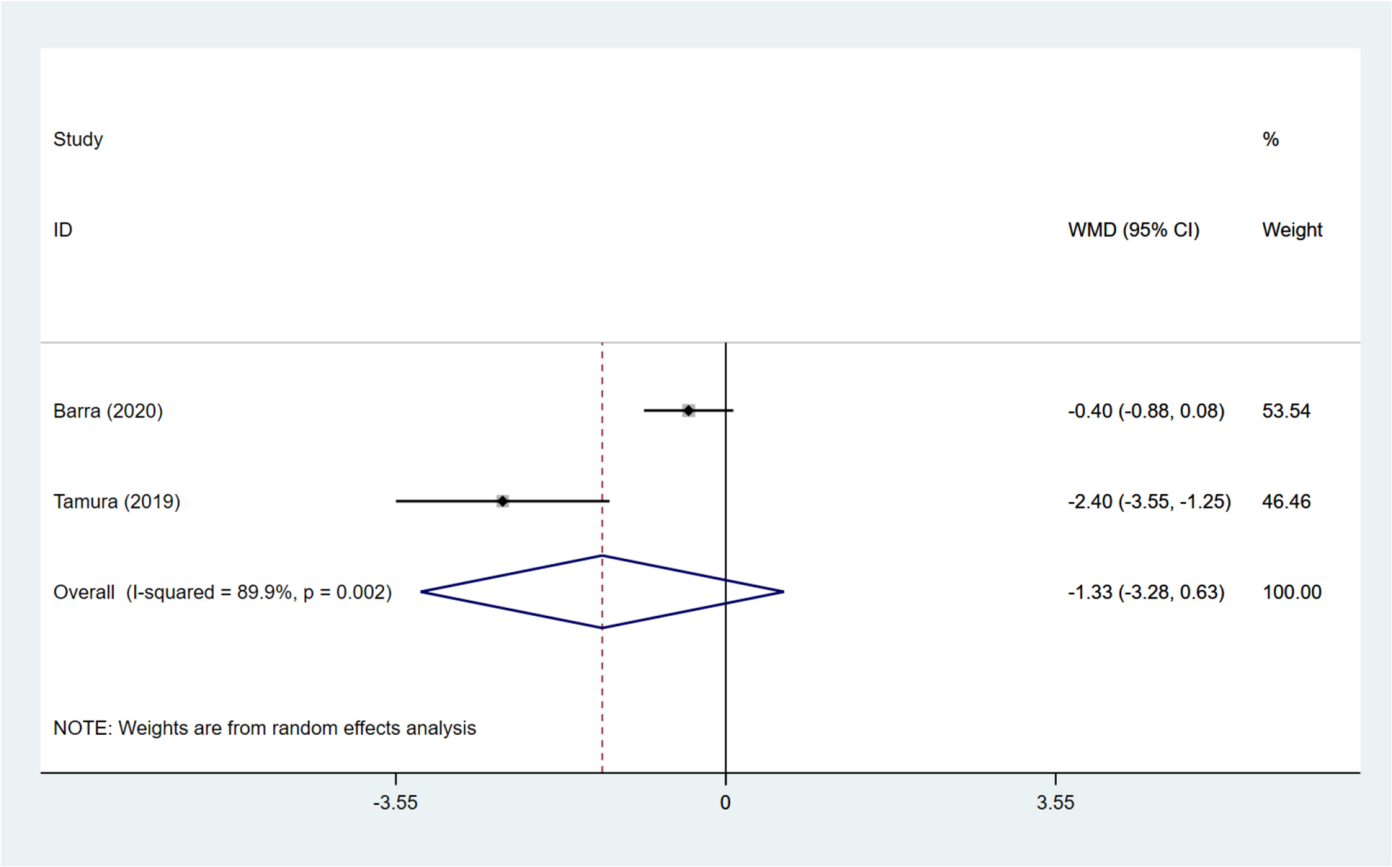
Forest plot for blastocysts in the DNG group versus non-DNG group DNG, dienogest; WMD, weighted mean difference; CI, confidence interval.

Two articles [12, 15] probed into the number of blastocysts following DNG treatment. DNG treatment was found to have an equivalent effect on the number of blastocysts to the non-DNG treatment (pooled WMD: -1.329, 95%CI: -3.284, 0.626, *P* = 0.183) (Fig. 7). Furthermore, subgroup analysis demonstrated no significant difference in the number of blastocysts between the DNG group and the non-hormonal treatment group (WMD: -0.400, 95%CI: -0.881, 0.081, *P* = 0.103), while the DNG group had significantly decreased blastocysts in contrast to the long GnRH-a group (WMD: -2.400, 95%CI: -3.549, -1.251, *P* < 0.001) (Table 3).

### Transferrable embryos

**Fig. 8:**
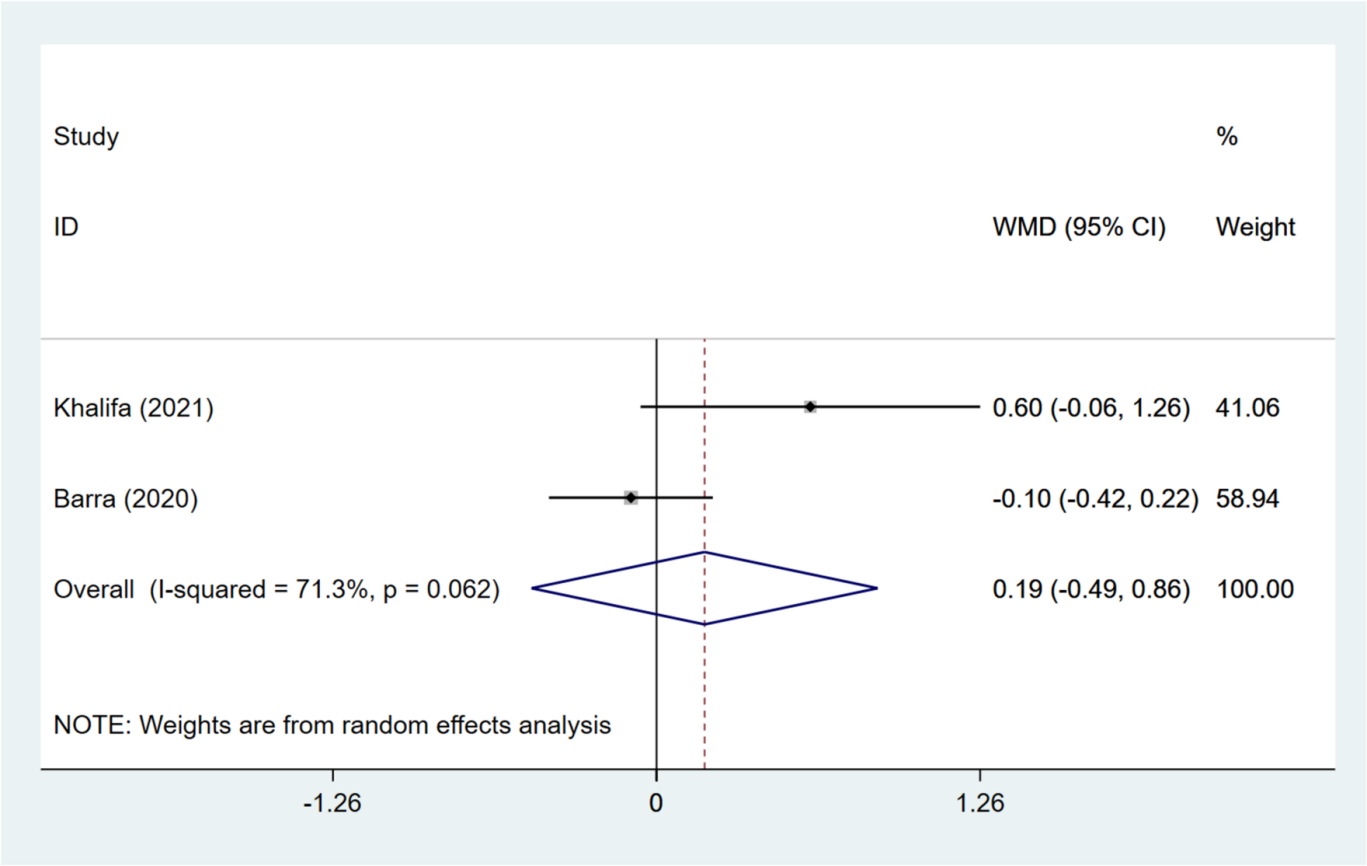
Forest plot for transferrable embryos in the DNG group versus non-DNG group DNG, dienogest; WMD, weighted mean difference; CI, confidence interval.

The number of transferrable embryos was evaluated in two publications [15, 17]. It was presented by combined analysis that DNG and non-DNG treatment exerted similar influences on the number of transferrable embryos (pooled WMD: 0.187, 95%CI: -0.487, 0.862, *P* = 0.586) (Fig. 8). Further, the number of transferrable embryos was comparable in the DNG and non-hormonal treatment groups (WMD: -0.100, 95%CI: -0.420, 0.220, *P* = 0.076), and in the DNG + short-acting GnRH-a and ultra-long GnRH-a groups (WMD: 0.600, 95%CI: -0.062, 1.262, *P* = 0.54) (Table 3).

### Fertilization rate

**Fig. 9:**
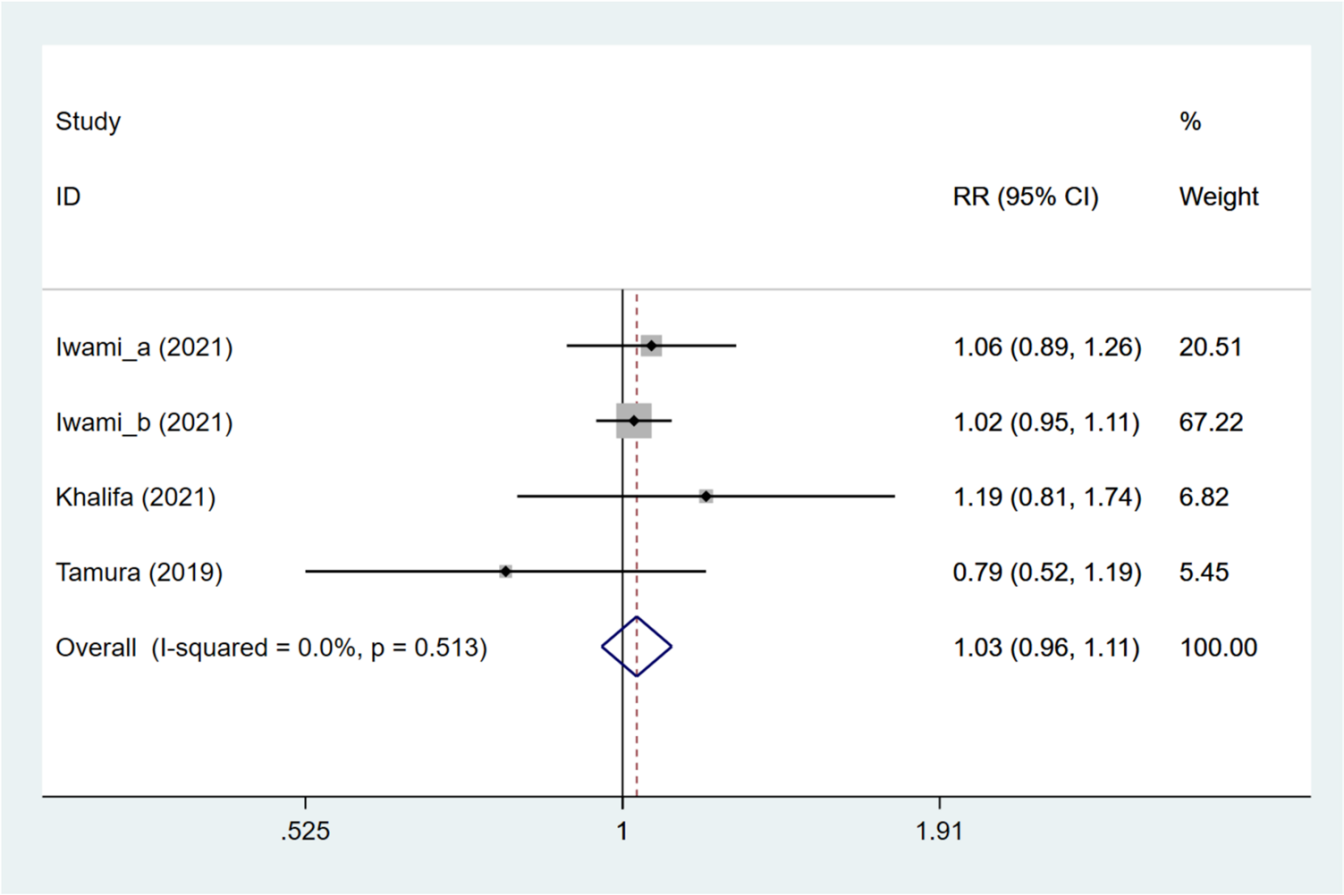
Forest plot for fertilization rate in the DNG group versus non-DNG group DNG, dienogest; RR, relative risk; CI, confidence interval.

Three articles [12, 16, 17] reported the fertilization rate of the DNG and non-DNG groups. Overall analysis showed that patients receiving DNG treatment had a similar fertilization rate to those without DNG treatment (pooled RR: 1.029, 95%CI: 0.958, 1.105, *P* = 0.429) (Fig. 9). Subgroup analysis found no significant difference in the fertilization rate between the DNG + short-acting GnRH-a and ultra-long GnRH-a groups (RR: 1.185, 95%CI: 0.807, 1.740, *P* = 0.386), between the DNG and long GnRH-a groups (RR: 0.788, 95%CI: 0.525, 1.185, *P* = 0.253), and between the DNG and dydrogesterone groups (pooled RR: 1.032, 95%CI: 0.961, 1.108, *P* = 0.386). Whether embryos transferred were frozen (pooled RR: 1.032, 95%CI: 0.961, 1.108, *P* = 0.386) or fresh (RR: 1.185, 95%CI: 0.807, 1.740, *P* = 0.386), the DNG and non-DNG treatment exhibited comparable effects on the fertilization rate (Table 3).

### Implantation rate

**Fig. 10:**
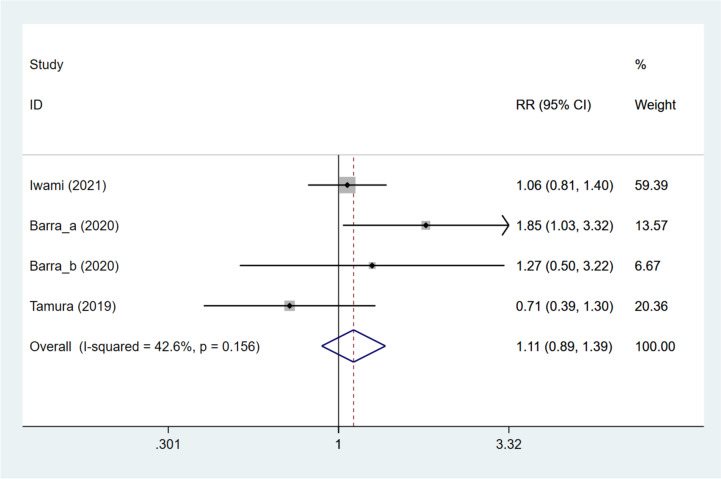
Forest plot for implantation rate in the DNG group versus non-DNG group DNG, dienogest; RR, relative risk; CI, confidence interval.

The implantation rate was measured in three studies [12, 15, 16]. No significant difference was identified between the DNG and non-DNG groups in the implantation rate (pooled RR: 1.111, 95%CI: 0.888, 1.391, *P* = 0.356) according to overall analysis (Fig. 10). In terms of different grouping methods, the DNG group had a notably increased implantation rate versus the non-hormonal treatment group (pooled RR: 1.657, 95%CI: 1.012, 2.715, *P* = 0.045), while the DNG and long GnRH-a groups (RR: 0.708, 95%CI: 0.387, 1.297, *P* = 0.264) as well as the DNG and dydrogesterone groups (RR: 1.064, 95%CI: 0.807, 1.403, *P* = 0.662) showed similar influences on the implantation rate. In term of embryo status, the implantation rate was equivalent in the DNG and non-DNG groups when using frozen embryos (pooled RR: 1.084, 95%CI: 0.830, 1.415, *P* = 0.553). While using fresh embryos, the DNG group had a significantly greater implantation rate than the non-DNG group (RR: 1.849, 95%CI: 1.031, 3.318, *P* = 0.039) (Table 3).

### Miscarriage rate

**Fig. 11:**
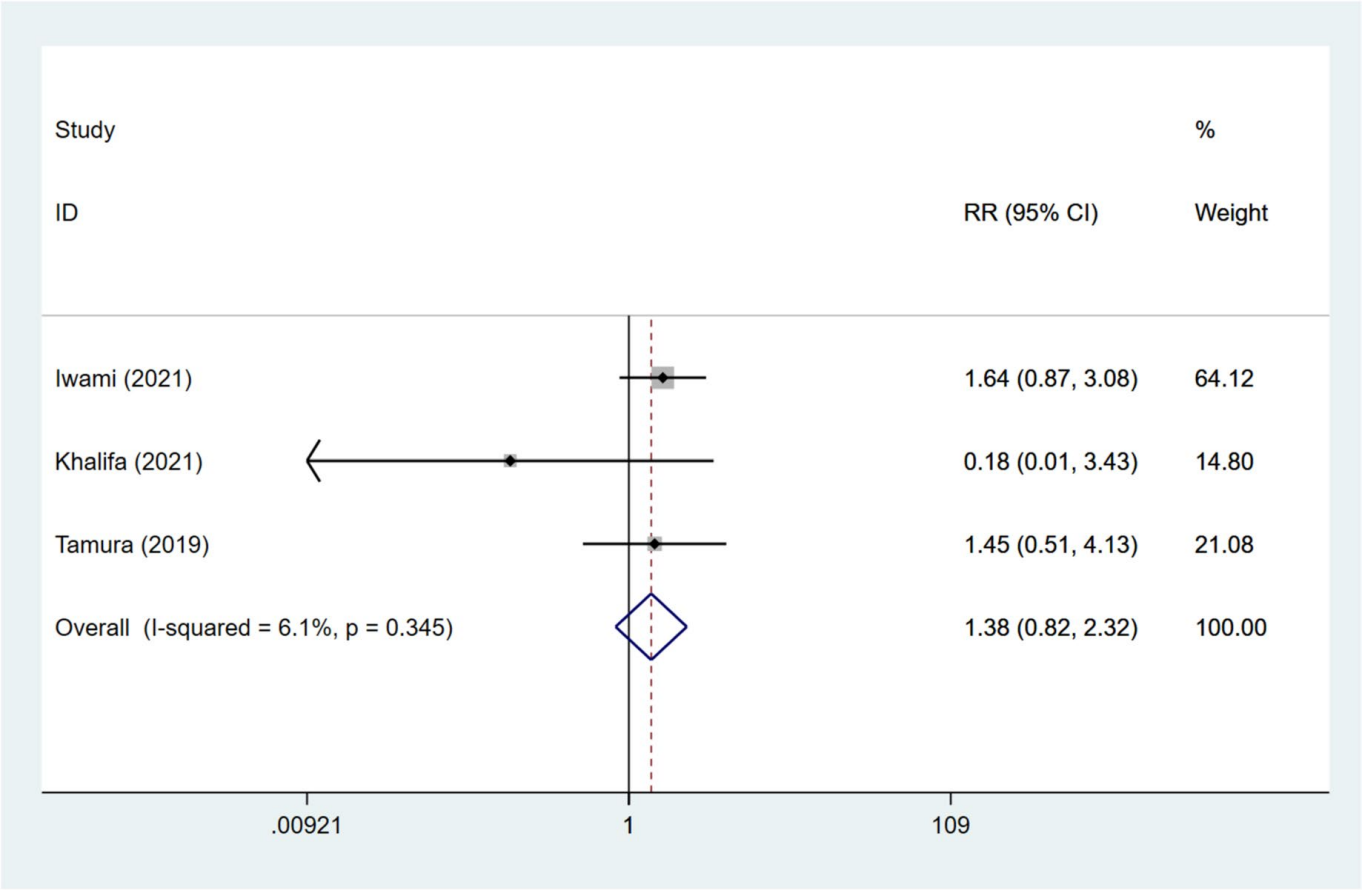
Forest plot for miscarriage rate in the DNG group versus non-DNG group DNG, dienogest; RR, relative risk; CI, confidence interval.

The impact of DNG treatment on the miscarriage rate was explored in three studies [12, 16, 17]. Combined analysis illustrated that no significant difference existed in the miscarriage rate between patients receiving DNG and non-DNG treatment (pooled RR: 1.384, 95%CI: 0.824, 2.325, *P* = 0.220) (Fig. 11). The DNG + short-acting GnRH-a and ultra-long GnRH-a groups (RR: 0.178, 95%CI: 0.009, 3.433, *P* = 0.253), the DNG and long GnRH-a groups (RR: 1.455, 95%CI: 0.512, 4.134, *P* = 0.482), as well as the DNG and dydrogesterone groups (RR: 1.639, 95%CI: 0.873, 3.078, *P* = 0.124) were identified to have comparable miscarriage rates, respectively. The miscarriage rate of the DNG group was similar to that of the non-DNG group regardless of embryo status (both *P* > 0.05) (Table 3).

### Sensitivity analysis

Sensitivity analysis was performed through removal of a study at a time and comprehensively analyzing the remaining studies. It was revealed that one-study removal did not significantly influence the combined results, indicating that the findings of this meta-analysis were stable and robust (Figures S1-S9).

## Discussion

This systematic review and meta-analysis compared DNG treatment with non-DNG treatment to assess the influence of DNG administration prior to IVF-ET on IVF-ET outcomes. Our findings demonstrated that DNG treatment exhibited similar effects to non-DNG treatment on either the primary or the secondary outcomes. According to subgroup analysis, the clinical pregnancy rate and live birth rate in the DNG group were significantly greater than those in the non-hormonal treatment group. Besides, DNG treatment was more effective for the clinical pregnancy rate and live birth rate than non-DNG treatment when patients underwent fresh embryo transfer.

DNG, a fourth-generation progestin, primarily used in endometriosis treatment, has been illustrated to be effective in relieving pain associated with the disease and well tolerated [18–21]. No significant difference was observed herein in IVF-ET outcomes between patients pretreated with DNG and not receiving DNG treatment before IVF-ET. Compared with non-hormonal treatment, DNG therapy obviously improved the clinical pregnancy rate and live birth rate among patients; DNG treatment was more effective than non-DNG treatment regarding the clinical pregnancy rate and live birth rate in patients using fresh embryos, which indicated that DNG treatment could be chosen over non-hormonal treatment in improving the clinical pregnancy rate and live birth rate after IVF-ET, and fresh embryos might be preferred for IVF-ET after DNG treatment. As regards the effect of long GnRH-a over DNG on the clinical pregnancy rate and live birth rate, only a RCT by Tamura et al. [12] was included for analysis. Progestin exerts influences on inhibiting follicular development and inducing follicle atresia [22, 23], and DNG can have the same influences [24, 25]. Administration of DNG preceding IVF-ET may thus lead to restrained follicle development and induced follicle atresia. Two included studies in this analysis showed that the number of growing follicles after DNG treatments was smaller than that after long GnRH-a and dydrogesterone treatments, respectively [12, 16]. Given the above side effects of DNG in suppressing follicle growth, more attention should be paid to the use of DNG, and future studies are needed to assess the impact of DNG pretreatment on IVF-ET outcomes among females with endometriosis and confirm our findings.

In terms of pharmacokinetics and pharmacodynamics, DNG has high oral bioavailability of over 90%, and short plasma half-life time of around 10 h, indicating no risk of accumulation after repeated administration and suitability of administration once a day [26]. DNG does not bind to the sex hormone binding globulin (SHBG) or corticoid binding globulin; thus, the application of DNG does not change the plasma levels of these proteins [20]. DNG has important progestational effects; it suppresses gonadotropic release, but does not have glucocorticoids, mineral corticoids or significant estrogen-like impacts in vivo [27, 28]. Although the affinity of DNG to progesterone receptors is low, it has an obvious progesterone influence in vivo, which can be resulted from the high levels of plasma free molecules [20]. Hence, DNG combines the advantages of 19-nortestosterone derivatives and progesterone derivatives. Compared with the activity of inhibiting ovulation, DNG has stronger activity on the endometrium. DNG-induced ovulation inhibition can be promptly retained after stopping treatment [24]. Since the levels of serum gonadotropins (FSH and luteinizing hormone [LH]) do not alter significantly, the effect on the ovary is peripheral rather than central. DNG is linked to a high incidence of abnormal menstrual bleeding patterns, but patients are generally well tolerated, with few discontinuing treatment, and the intensity and frequency of bleeding decline with time [29].

DNG can effectively relieve pain symptoms related to endometriosis, like dysmenorrhea, premenstrual pelvic pain, dyspareunia and chronic pelvic pain. Its efficacy is better than that of placebo and comparable to that of GnRH-a, but with better tolerance. Its great endometrial efficacy makes it anti-proliferative and anti-inflammatory in treating endometriotic lesions [26]. DNG stimulates the differentiation of endometrial stromal cells (ESC) and suppresses their proliferation [30]. DNG suppresses aromatase and COX-2 expression as well as prostaglandin E2 production in ESC in an experimental in vitro study. These pharmacological characteristics may facilitate the therapeutic effect of DNG on endometriosis, thereby exhibiting the significant anti-inflammatory impact of DNG associated with size reduction of endometrial lesions [9, 31]. Evidence demonstrated that DNG had a great impact on the inflammatory microenvironment of endometrial lesions, which may promote its clinical efficacy [30]. DNG exhibits favorable impacts on systemic and intralesional inflammatory microenvironments for females who have endometriosis. It reduces secretion of interleukin-8 (IL-8), IL-6 and monocyte chemotactic protein-1 (MCP-1), and lowers TNF-α-induced generation of mRNA in endometrial stromal cells from these females [32]. Furthermore, the antigen-presenting function of peritoneal fluid macrophages can be recovered by DNG via upregulating human leucocyte antigen (HLA)-DR [33]. DNG was reported to have a favorable impact at the endometrial level [34], and among these females, the eutopic endometrium response to steroid hormones may be damaged by aberrant expression of estrogen receptors (ER) and progesterone receptors (PR) [35], causing “progesterone-resistant” condition. Blocked endometrial secretory conversion, implantation failure, or its pathological change following ET may be due to this [36]. A recent investigation by Hayashi et al. [34] illustrated that DNG might ameliorate the progesterone resistance in endometrial tissue through elevating the PR-B/PR-A ratio and reducing the ERβ/ERα isoform ratio, so it might positively affect pregnancy outcomes. One reason of DNG’s effectiveness in endometriosis is that DNG creates a hypoestrogenic situation at the endometrial tissue level, but does not excessively reduce the plasma E2 concentration, which is often stable at the lower limit of the normal concentration range [37]. Compared with GnRH analogues, such E2 levels are not expected to cause the reactivation of endometriotic lesions, whereas they are high enough to prevent hot flashes and bone loss, which has been found during the treatment of endometriosis with DNG [38]. DNG has low androgen receptor activity and some antiandrogenic activity [39], which explains the limited androgen-like side effects, such as weight gain, acne, alopecia and hirsutism [20]. Side effects caused by hypoestrogenism (such as hot flashes and bone loss) do not occur, whereas these effects are found during the treatment of endometriosis with GnRH analogues [38].

Despite efficiently relieved pain and reduced development of endometriotic implants [40], GnRH-a is relevant to hypoestrogenic adverse effects, including hot flushes, headaches, vaginal dryness, decreased libido, and loss of bone mineral density [34, 41]. Because of these side effects, many patients “keep in mind” GnRH-a treatment and do not redo it all the time, suggesting a decline in adherence to the treatment. Given this, DNG could be prescribed as an alternative owing to no significant antigonadotropin impact and remarkably inhibited ovarian function. Further, DNG is suggested to have greater adherence and, as illustrated by this study, may contribute to increasing clinical pregnancy and live birth rates. Besides, decreased bone mineral density usually limits the longest duration of treatment to 3–6 months, unless low-dose, “add-back” therapy with an estrogen, a progestogen or an estrogen/progestogen combination is added to reduce hypoestrogenic adverse effects [41, 42]. Although add-back therapy can prevent bone density loss and allow GnRH-a to be applied for longer time, it greatly increases the cost and complexity of treatment [29, 42].

The bioavailability of dydrogesterone in human is low. The plasma concentration of dydrogesterone was lower than the detection limit (< 1 ng/mL), while after oral administration of 10 mg dydrogesterone, the metabolite among women was approximately 10 ng/mL. Some metabolites of dydrogesterone may facilitate progesterone activity in vivo [39]. A premature LH surge and partial ovulation can compromise oocyte yields and lower the pregnancy rate [43]. Dydrogesterone, an efficacious oral substitute for GnRH agonists or antagonists, is generally utilized to prevent premature LH surge in females receiving controlled ovarian hyperstimulation (COH) [44, 45]. Progestin-primed ovarian stimulation (PPOS) using DNG has several underlying advantages over PPOS using dydrogesterone, comprising anti-inflammatory effects, endometriosis relapse suppression after surgical intervention, and endometriosis-associated pain relief [16]. DNG has good specificity for the progesterone receptor in contrast to dydrogesterone [39], has a direct inhibiting influence on the proliferation of endometrial lesions, and has a stronger cytoreductive impact on endometrial lesions than GnRH-a [46–48]. Additionally, DNG is cheaper than GnRH-a and can be taken orally, while GnRH-a must be administered subcutaneously or nasally [16]. Different from natural GnRH, this substitution makes the agonist resistant to degradation by endopeptidases and makes its half-life longer, thus prolonging receptor occupancy [49].

Based on the above, pretreatment with DNG might be administrable to patients with endometriosis undergoing IVF-ET. In clinical practice, DNG could be administered over non-hormonal treatment to improve the clinical pregnancy rate and live birth rate. Importantly, this drug might be used alone with caution, since the clinical pregnancy and live birth rates following long GnRH-a treatment were shown to be greater than those after DNG use [12], and the dydrogesterone group displayed the elevated number of mature oocytes versus the DNG group [16], for which more research is warranted. Besides, patients receiving DNG combined with short-acting GnRH-a showed fewer mature oocytes than those receiving ultra-long GnRH-a [17], indicating that the combined medication of DNG needs further clinical validation.

There were some limitations in the current study: for one thing, the amount of eligible literature for analysis was relatively small, and more clinical studies are required to ensure the stability of results; for another, not all included studies reported the stage of the disease, the kind of surgery the patients had, and whether patients had removal of endometriomas or adhesions or bowel resection prior to IVF. No included studies reported whether the patients had previous GnRH antagonist treatments prior to the current treatment with dianogest. Four (out of five studies) excluded patients with long-term hormone therapy for endometriosis, and one study [11] focused on the population planning to undergo IVF following laparoscopic removal of endometriomas, with no endometriomas or other ovarian cysts at the start of stimulation. Based on the above, subgroup analysis could not be performed based on these factors to improve the purity of results. Reporting of the factors related to results should be improved in future studies. For great heterogeneity, we also attempted to explore the source of heterogeneity via meta-regression analysis. The results showed that meta-regression analysis could only be performed on the outcomes clinical pregnancy and live birth due to the limited number of eligible studies for analysis, and the heterogeneity in clinical pregnancy and live birth did not come from grouping methods and embryo types. More studies are needed to assess the source of heterogeneity. It is also indicated that studies to be conducted should pay attention to the selection of the study population to make the enrolled data homogeneous, in order to better evaluate the influence of interventions on outcomes. Future well-designed studies are warranted to support our findings.

## Conclusion

DNG treatment may have comparable effects to non-DNG treatment on IVF-ET outcomes. The clinical pregnancy rate and live birth rate in the DNG group may be significantly greater than those in the non-hormonal treatment group. These findings indicated that DNG may be administrable over non-hormonal treatment to improve the clinical pregnancy rate and live birth rate, and this drug might be used alone with caution for patients with endometriosis who would undergo IVF-ET. More investigations are underscored to confirm our findings.

### Supplementary Information

**Additional file 1: Figure S1.** Sensitivity analysis for clinical pregnancy rate in the DNG group versus non-DNG group. DNG, dienogest; CI, confidence interval.

**Additional file 2: Figure S2.** Sensitivity analysis for live birth rate in the DNG group versus non-DNG group. DNG, dienogest; CI, confidence interval.

**Additional file 3: Figure S3.** Sensitivity analysis for retrieved oocytes in the DNG group versus non-DNG group. DNG, dienogest; CI, confidence interval.

**Additional file 4: Figure S4.** Sensitivity analysis for mature oocytes in the DNG group versus non-DNG group. DNG, dienogest; CI, confidence interval.

**Additional file 5: Figure S5.** Sensitivity analysis for blastocysts in the DNG group versus non-DNG group. DNG, dienogest; CI, confidence interval.

**Additional file 6: Figure S6.** Sensitivity analysis for transferrable embryos in the DNG group versus non-DNG group. DNG, dienogest; CI, confidence interval.

**Additional file 7: Figure S7.** Sensitivity analysis for fertilization rate in the DNG group versus non-DNG group. DNG, dienogest; CI, confidence interval.

**Additional file 8: Figure S8.** Sensitivity analysis for implantation rate in the DNG group versus non-DNG group. DNG, dienogest; CI, confidence interval.

**Additional file 9: Figure S9.** Sensitivity analysis for miscarriage rate in the DNG group versus non-DNG group. DNG, dienogest; CI, confidence interval.

**Additional file 10: Table S1.** Meta-regression analysis for clinical pregnancy and live birth rates in the DNG group versus non-DNG group. DNG, dienogest; GnRH-a, gonadotropin-releasing hormone agonist; Coef, coefficient; Std. Err, standard error; CI, confidence interval.

## Data Availability

The datasets used and/or analyzed during the current study are available from the corresponding author on reasonable request.

## Abbreviations

ART: Assisted reproduction technology
IVF-ET: In vitro fertilization and embryo transfer
GnRH-a: Gonadotropin-releasing hormone agonist
DNG: Dienogest
BMI: Body mass index
FSH: Follicle-stimulating hormone
AMH: Anti-Mullerian hormone
AFC: Antral follicle count
NOS: Newcastle–Ottawa Scale
RRs: Relative risks
WMDs: Weighted mean differences
CIs: Confidence intervals
