# Supplementary figures and images for: Impact of dienogest pretreatment on IVF-ET outcomes in patients with endometriosis: a systematic review and meta-analysis
Source: J Ovarian Res. 2023 Aug 16;16:166. doi: 10.1186/s13048-023-01245-8 (PMC10428538; doi:10.1186/s13048-023-01245-8)

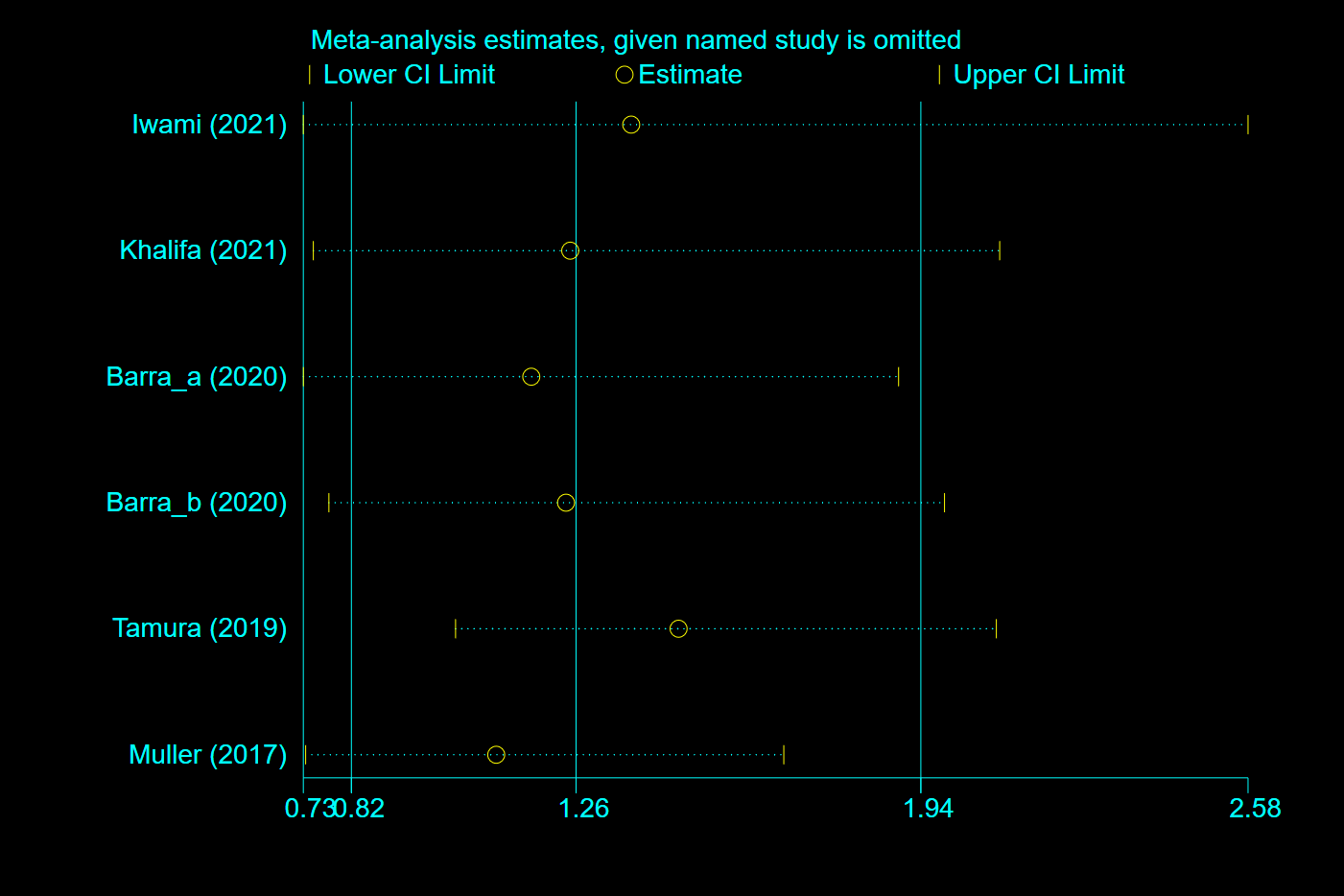

Supplement: Supplementary file 1 — Additional file 1: Figure S1. Sensitivity analysis for clinical pregnancy rate in the DNG group versus non-DNG group. DNG, dienogest; CI, confidence interval. [file 13048_2023_1245_MOESM1_ESM.tif]

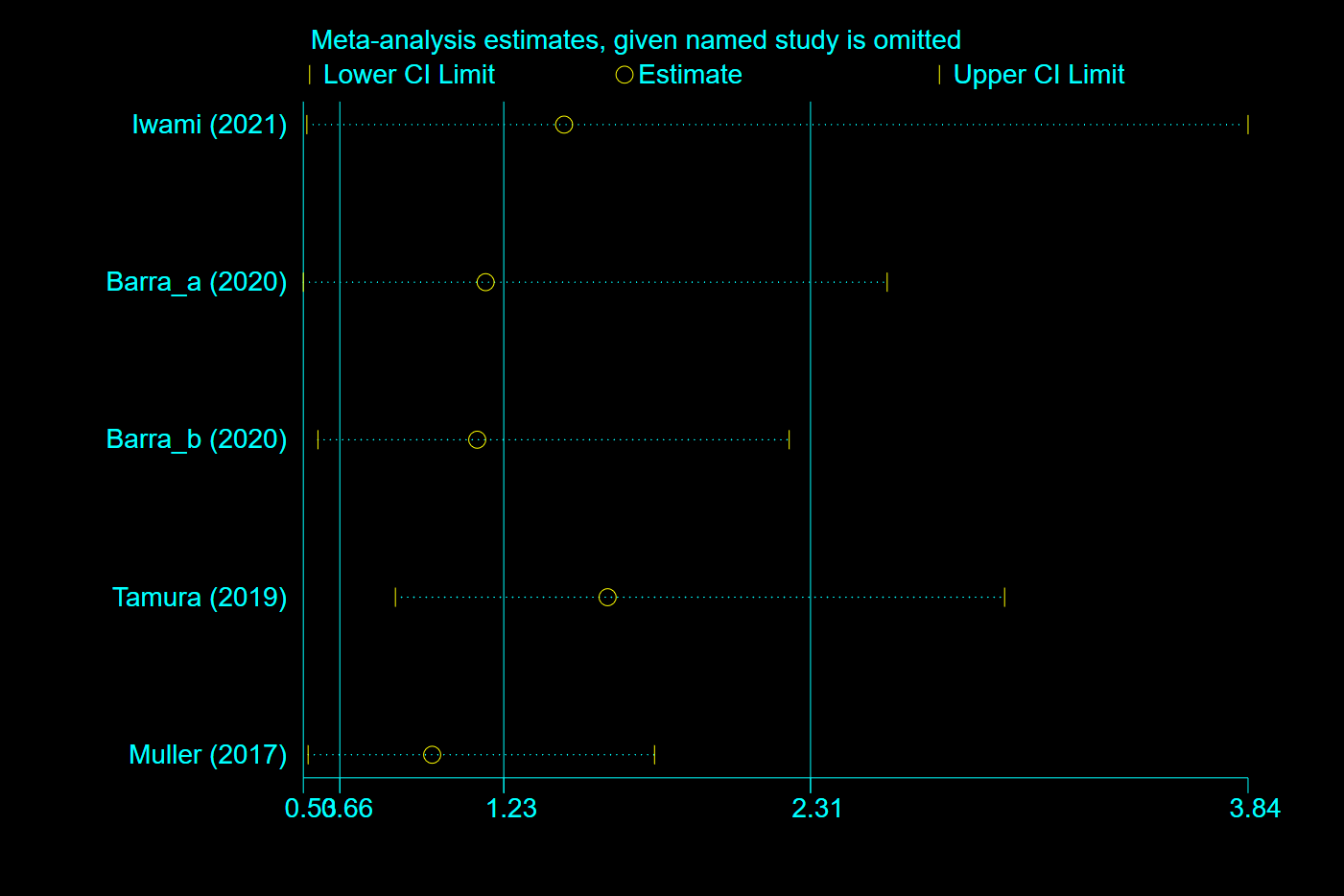

Supplement: Supplementary file 2 — Additional file 2: Figure S2. Sensitivity analysis for live birth rate in the DNG group versus non-DNG group. DNG, dienogest; CI, confidence interval. [file 13048_2023_1245_MOESM2_ESM.tif]

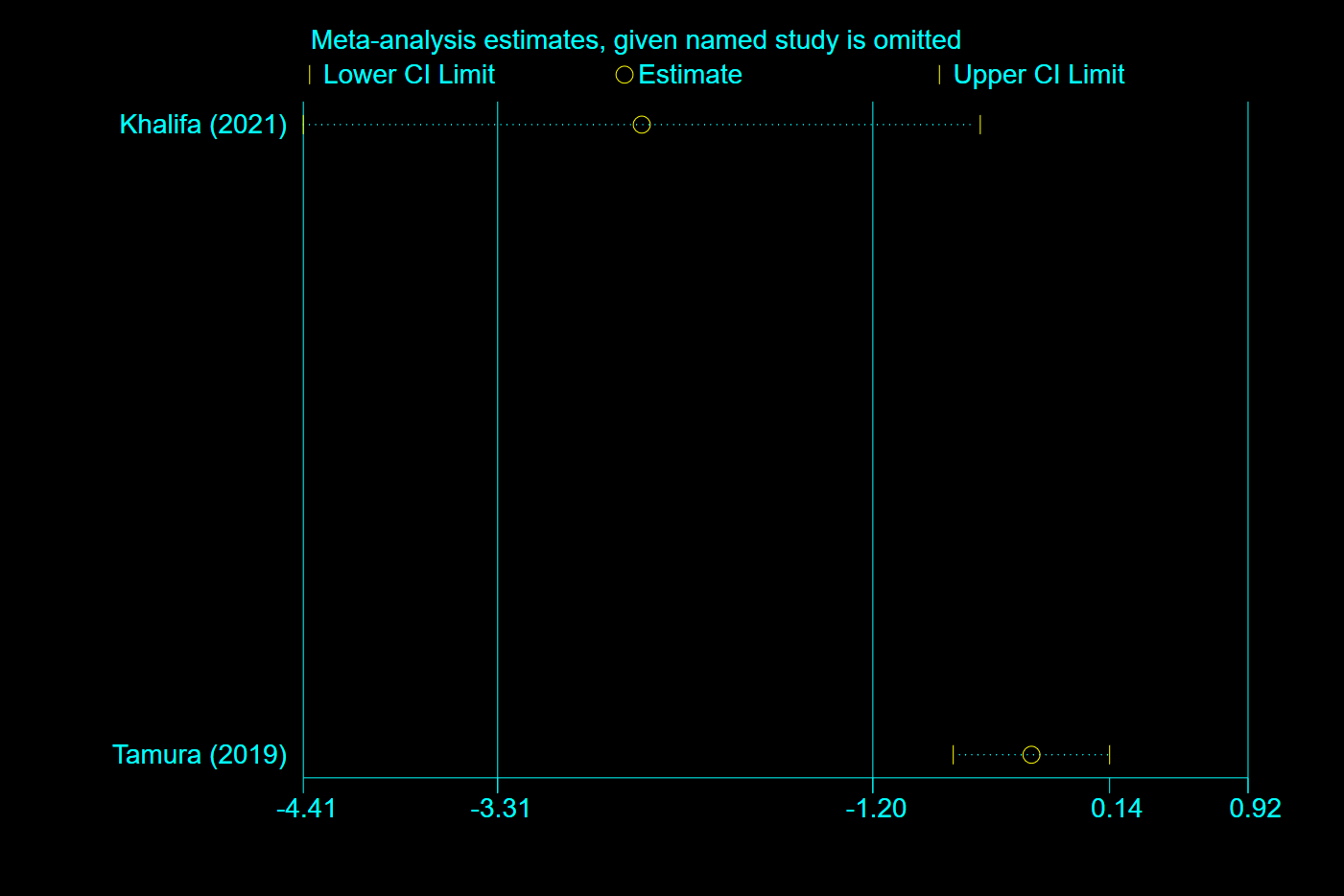

Supplement: Supplementary file 3 — Additional file 3: Figure S3. Sensitivity analysis for retrieved oocytes in the DNG group versus non-DNG group. DNG, dienogest; CI, confidence interval. [file 13048_2023_1245_MOESM3_ESM.tif]

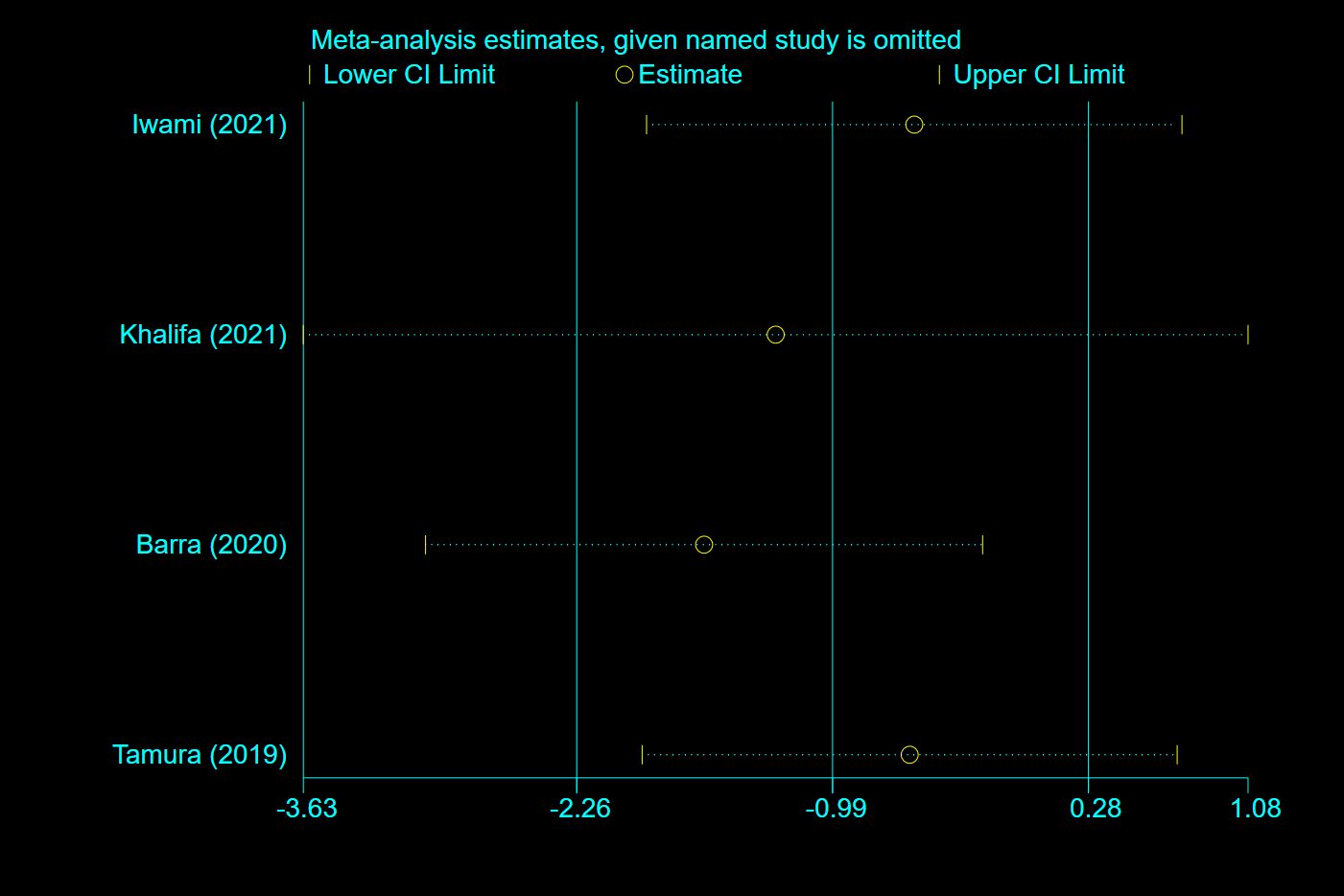

Supplement: Supplementary file 4 — Additional file 4: Figure S4. Sensitivity analysis for mature oocytes in the DNG group versus non-DNG group. DNG, dienogest; CI, confidence interval. [file 13048_2023_1245_MOESM4_ESM.tif]

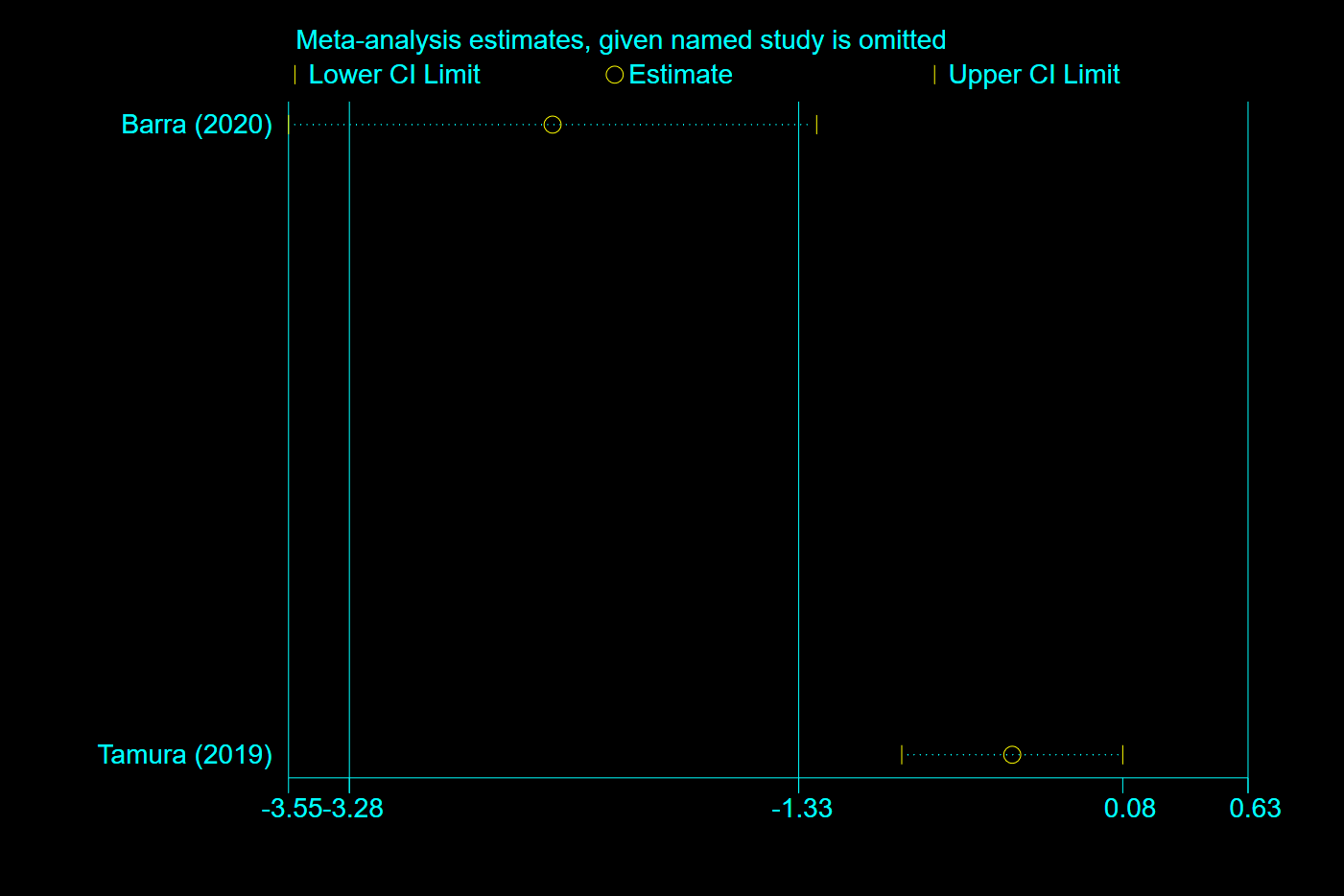

Supplement: Supplementary file 5 — Additional file 5: Figure S5. Sensitivity analysis for blastocysts in the DNG group versus non-DNG group. DNG, dienogest; CI, confidence interval. [file 13048_2023_1245_MOESM5_ESM.tif]

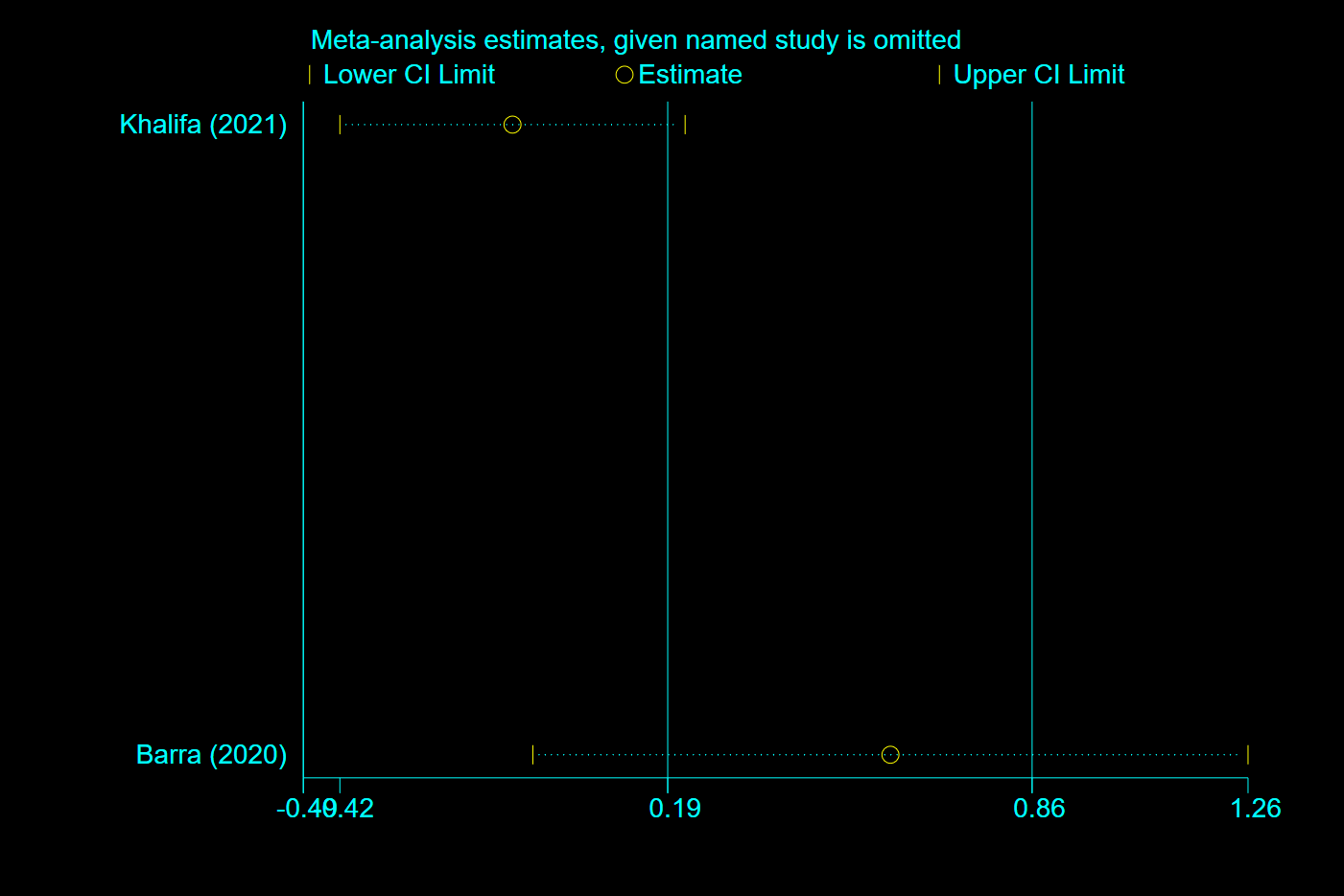

Supplement: Supplementary file 6 — Additional file 6: Figure S6. Sensitivity analysis for transferrable embryos in the DNG group versus non-DNG group. DNG, dienogest; CI, confidence interval. [file 13048_2023_1245_MOESM6_ESM.tif]

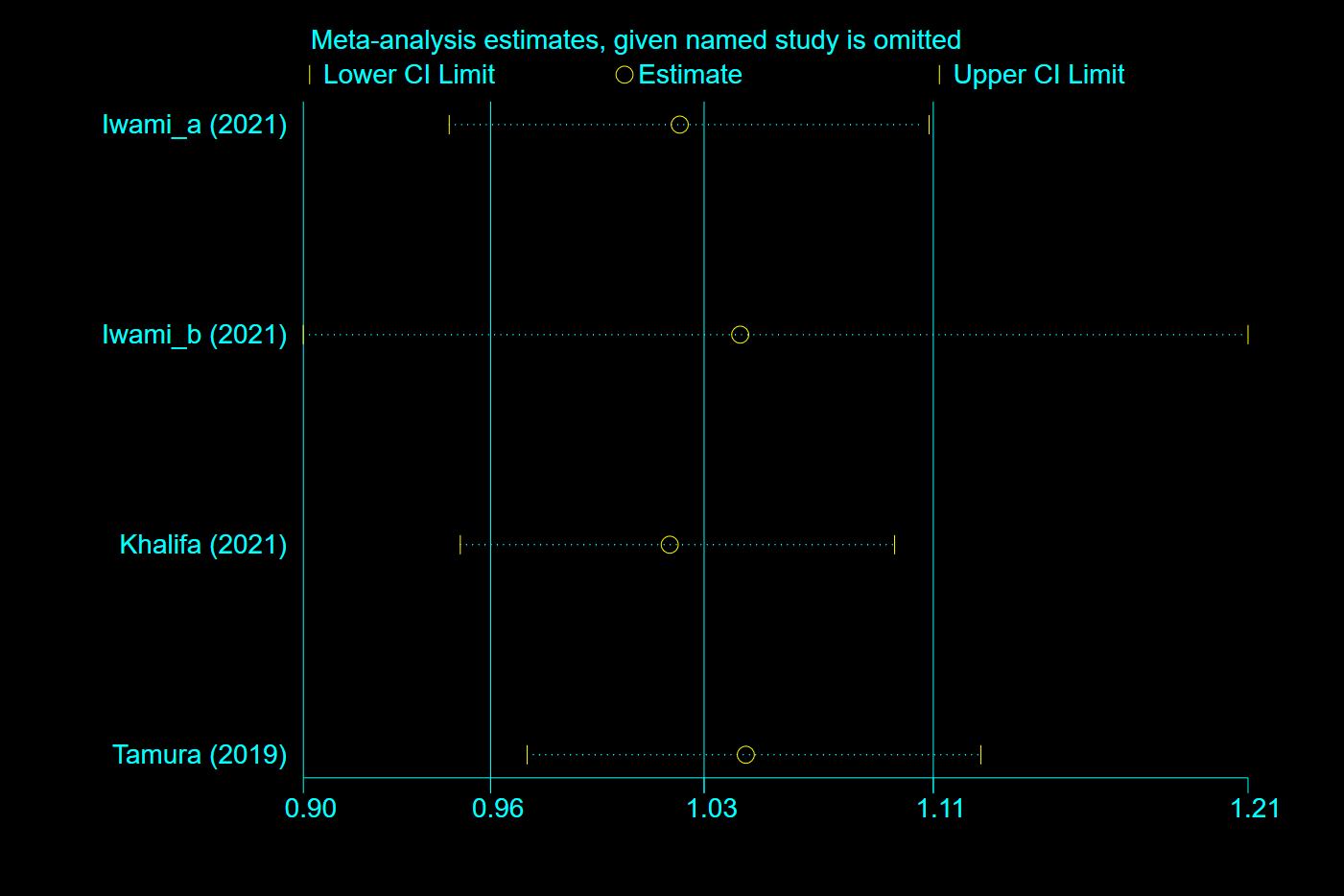

Supplement: Supplementary file 7 — Additional file 7: Figure S7. Sensitivity analysis for fertilization rate in the DNG group versus non-DNG group. DNG, dienogest; CI, confidence interval. [file 13048_2023_1245_MOESM7_ESM.tif]

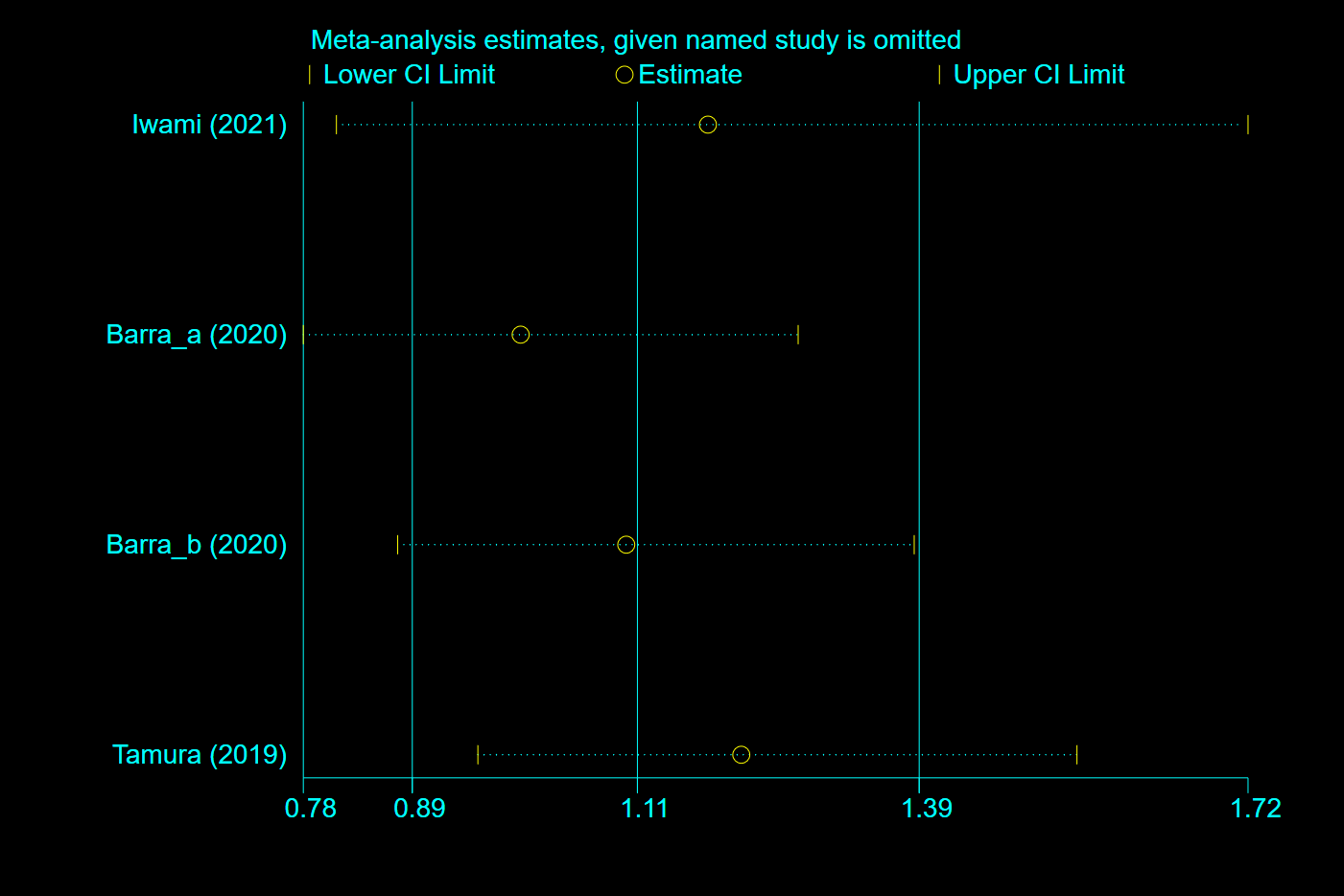

Supplement: Supplementary file 8 — Additional file 8: Figure S8. Sensitivity analysis for implantation rate in the DNG group versus non-DNG group. DNG, dienogest; CI, confidence interval. [file 13048_2023_1245_MOESM8_ESM.tif]

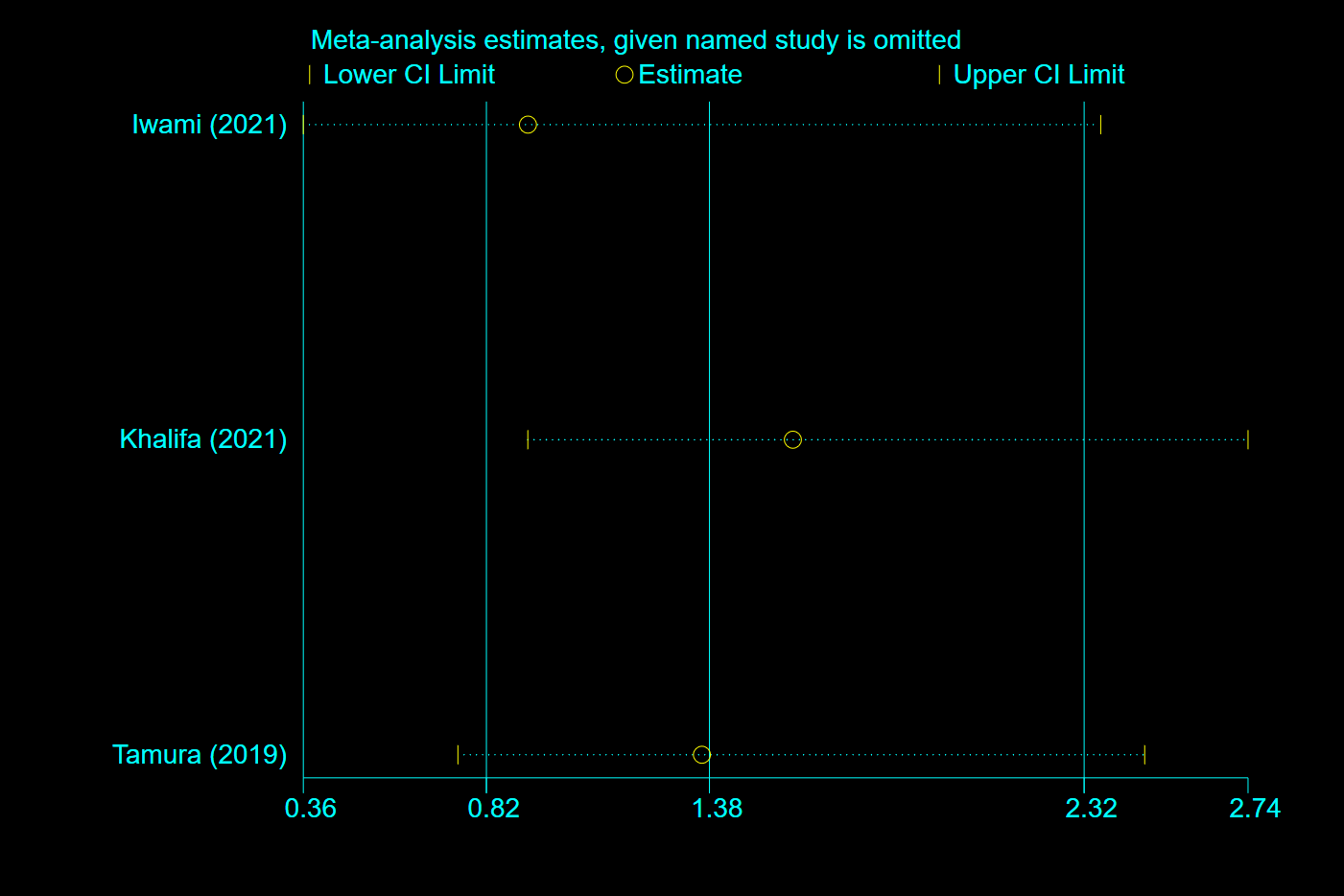

Supplement: Supplementary file 9 — Additional file 9: Figure S9. Sensitivity analysis for miscarriage rate in the DNG group versus non-DNG group. DNG, dienogest; CI, confidence interval. [file 13048_2023_1245_MOESM9_ESM.tif]
