# Supplementary material for: Impact of dienogest pretreatment on IVF-ET outcomes in patients with endometriosis: a systematic review and meta-analysis
Source: J Ovarian Res. 2023 Aug 16;16:166. doi: 10.1186/s13048-023-01245-8 (PMC10428538; doi:10.1186/s13048-023-01245-8)
Supplement: Supplementary file 10 — Additional file 10: Table S1. Meta-regression analysis for clinical pregnancy and live birth rates in the DNG group versus non-DNG group. DNG, dienogest; GnRH-a, gonadotropin-releasing hormone agonist; Coef, coefficient; Std. Err, standard error; CI, confidence interval. [file 13048_2023_1245_MOESM10_ESM.docx]

**Table S1.** Meta-regression analysis for clinical pregnancy and live birth rates in the DNG group versus non-DNG group.

| Variable | Coef | Std. Err | t | *P* | 95%CI | |
| --- | --- | --- | --- | --- | --- | --- |
| Clinical pregnancy rate |  |  |  |  |  |  |
| Group |  |  |  |  |  |  |
| (DNG vs. long GnRH-a) vs. (DNG vs. no treatment) | -1.552 | 0.546 | -2.84 | 0.105 | -3.901 | 0.798 |
| (DNG+ short-acting GnRH-a vs. ultra-long GnRH-a) vs. (DNG vs. no treatment) | -0.677 | 0.582 | -1.16 | 0.365 | -3.182 | 1.828 |
| (DNG vs. dydrogesterone) vs. (DNG vs. no treatment) | -1.016 | 0.495 | -2.05 | 0.176 | -3.144 | 1.113 |
| Constant | 2.094 | 0.324 | 6.47 | 0.023 | 0.700 | 3.487 |
| Embryo |  |  |  |  |  |  |
| Frozen vs. fresh | -0.737 | 0.489 | -1.51 | 0.229 | -2.293 | 0.819 |
| Constant | 1.941 | 0.316 | 6.15 | 0.009 | 0.936 | 2.946 |
| Live birth rate |  |  |  |  |  |  |
| Group |  |  |  |  |  |  |
| (DNG vs. long GnRH-a) vs. (DNG vs. no treatment) | -2.001 | 1.034 | -1.94 | 0.193 | -6.448 | 2.447 |
| (DNG vs. dydrogesterone) vs. (DNG vs. no treatment) | -1.499 | 0.983 | -1.53 | 0.267 | -5.727 | 2.728 |
| Constant | 2.442 | 0.559 | 4.37 | 0.049 | 0.038 | 4.845 |
| Embryo |  |  |  |  |  |  |
| Frozen vs. fresh | -0.881 | 1.146 | -0.77 | 0.523 | -5.812 | 4.051 |
| Constant | 2.436 | 0.797 | 3.06 | 0.092 | -0.994 | 5.866 |

DNG, dienogest; GnRH-a, gonadotropin-releasing hormone agonist; Coef, coefficient; Std. Err, standard error; CI, confidence interval.
